# Bach2–Batf interactions control Th2-type immune response by regulating the IL-4 amplification loop

**DOI:** 10.1038/ncomms12596

**Published:** 2016-09-01

**Authors:** Makoto Kuwahara, Wataru Ise, Mizuki Ochi, Junpei Suzuki, Kohei Kometani, Saho Maruyama, Maya Izumoto, Akira Matsumoto, Nobuaki Takemori, Ayako Takemori, Kenta Shinoda, Toshinori Nakayama, Osamu Ohara, Masaki Yasukawa, Tatsuya Sawasaki, Tomohiro Kurosaki, Masakatsu Yamashita

**Affiliations:** 1 Department of Immunology, Graduate School of Medicine, Ehime University, Shitsukawa, Toon, Ehime 791-0295, Japan; 2 Department of Translational Immunology, Translational Research Center, Ehime University Hospital, Shitsukawa, Toon, Ehime 791-0295, Japan; 3 Division of Immune Regulation, Department of Proteo-Inovation, Proteo-Science Center, Ehime University, Toon, Ehime 791-0295, Japan; 4 Department of Lymphocyte Differentiation, WPI Immunology Frontier Research Center, Osaka University, 3-1 Yamada-oka, Suita, Osaka 565-0871, Japan; 5 Division of Cell-Free Sciences, Department of Proteo-Research, Proteo-Science Center, Ehime University, Matsuyama, Ehime 790-8577, Japan; 6 Department of Hematology, Clinical Immunology and Infectious Diseases, Graduate School of Medicine, Ehime University, Shitsukawa, Toon, Ehime 791-0295, Japan; 7 Laboratory for Lymphocyte Differentiation, RIKEN Center for Integrative Medical Sciences, 1-7-22 suehiro-cho, Tsurumi-ku, Yokohama 230-0045, Japan; 8 Department of Infection and Host Defenses, Graduate School of Medicine, Ehime University, Shitsukawa, Toon, Ehime 791-0295, Japan; 9 Division of Proteomics, Department of Proteo-Medicine, Proteo-Science Center, Ehime University, Toon, Ehime 791-0295, Japan; 10 Department of Immunology, Graduate School of Medicine, Chiba University, 1-8-1 Inohana, Chuo-ku, Chiba 260-8670 Japan; 11 Human DNA Analysis Group, Department of Technology Development, Kazusa DNA Research Institute, 2-6-7 Kazusa-Kamatari, Kisarazu, Chiba 292-0818, Japan

## Abstract

Although Bach2 has an important role in regulating the Th2-type immune response, the underlying molecular mechanisms remain unclear. We herein demonstrate that Bach2 associates with Batf and binds to the regulatory regions of the Th2 cytokine gene loci. The Bach2–Batf complex antagonizes the recruitment of the Batf–Irf4 complex to AP-1 motifs and suppresses Th2 cytokine production. Furthermore, we find that Bach2 regulates the *Batf* and *Batf3* expressions via two distinct pathways. First, Bach2 suppresses the maintenance of the *Batf* and *Batf3* expression through the inhibition of IL-4 production. Second, the Bach2–Batf complex directly binds to the *Batf* and *Batf3* gene loci and reduces transcription by interfering with the Batf–Irf4 complex. These findings suggest that IL-4 and Batf form a positive feedback amplification loop to induce Th2 cell differentiation and the subsequent Th2-type immune response, and Bach2–Batf interactions are required to prevent an excessive Th2 response.

## Abstract (web summary)

Bach2 limits T cell effector functions. Here the authors show that Bach2–Batf complex antagonizes the recruitment of the Batf–Irf4 complex to AP-1 motifs and suppresses Th2 cytokine production, and describe mechanisms of negative feedback by which Bach2 restricts Baft-mediated Th2 response.

## Graphical abstract

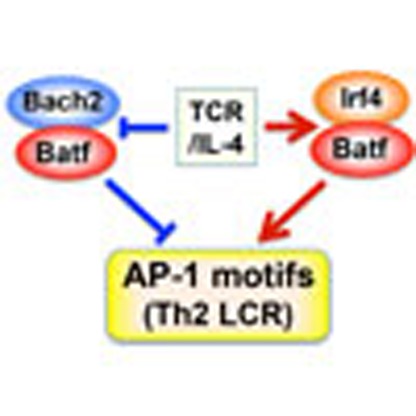

Elucidating the molecular mechanisms by which naive CD4 T cells differentiate into effector helper T (Th) cells is important for understanding T cell-mediated immune responses. Functionally distinct Th subsets have been reported, including Th1, Th2, Th17 and inducible regulatory T (iTreg) cells^1^,^2^,^3^,^4^,^5^,^6^. Several transcription factors that control the differentiation of these Th subsets have been identified such as T-bet, Gata3, Rorγt and Foxp3 for Th1, Th2, Th17 and iTreg cells, respectively^1^,^2^,^3^,^4^,^5^,^6^.

The murine Th2 cytokine genes encoding interleukin (IL)-4, IL-5 and IL-13 are located within a 140-kb region on chromosome 11 flanking the *Rad50* genes^7^. The locus control region (LCR) for the Th2 cytokine gene loci has been mapped to a region of ∼25-kb within the 3′ intronic regions of the *Rad50* genes^8^. DNA hypersensitivity analyses have revealed the presence of several evolutionally conserved hypersensitive sites, named Rad50 hypersensitive site (RHS4–7; ref. 8). The intron 2 region of the *Il4* gene (DNase I hypersensitive-site 2: HS2, *Il4* IE), a Gata3-binding site, is crucial for the production of IL-4 by CD4 T cells^9^, and the deletion of the *Il4* IE site result in the reduction of IL-4 production, but not that of IL-5 or IL-13, in Th2 cells. The conserved Gata3-response element (CGRE) upstream of the *Il13* gene locus is important to control widespread chromatin modifications of the *Il13* and *Il4* gene loci^10^, and the deletion of CGRE site is resulted in the reduced generation of IL-13-producing Th2 cells^9^.

BTB and Cap‘n'collar (CNC) homology 1; basic leucine zipper transcription factor 2 (Bach2) belongs to the CNC gene family^11^. B cells preferentially express Bach2, which is critical for somatic hypermutation and class-switch recombination^13^,^14^,^15^, and is involved in the IgG1 memory B cell formation^16^. A recent report by Itoh-Nakadai *et al*.^17^ demonstrated that Bach2 promotes B cell development via the repression of the myeloid program^18^. Bach2 also participates in T-cell-mediated immune responses and regulates T_reg_-mediated immune homeostasis, and *Bach2* null animals suffer from lethal lung and small intestinal inflammation^19^,^20^. Bach2 is required for the maintenance of naive CD4 T cells by suppressing the effector memory-related gene expression^21^. In addition, an important role of Bach2 in the memory CD8 T cell generation was reported^22^. We recently demonstrated that senescence-associated secretory phenotype is rapidly induced in *Bach2*-deficient activated CD4 T cells^23^. Although these findings establish Bach2 as a key regulator of CD4 T cell-mediated immune homeostasis, the molecular mechanism(s) by which Bach2 controls the CD4 T cell function and differentiation has yet to be fully elucidated.

Basic leucine zipper transcription factor ATF-like (Batf) family transcription factors belong to the activator protein 1 (AP-1) family of transcription factors, which regulate various cellular processes^24^. The Batf family comprises three transcription factors, Batf, Batf2 and Batf3, which are composed of only a DNA-binding domain and a leucine zipper motif that were originally identified as inhibitors of AP-1-dependent transcriptional activation^25^,^26^. However, a recent study revealed that Batfs interact with interferon-regulatory factor (Irf) family proteins including Irf4, and activate Irf-mediated transcription^27^,^28^. Batf family transcription factors are involved in the regulation of helper T cell subset differentiation^24^. Batf is required for the differentiation and functions of Th2, Th17 and Tfh cells, and Batf3 regulates the Th2 cell function. In contrast, Batf and Batf3 are not essential for the interferon-γ (IFN-γ) expression in Th1 cells.

In this study, we found that Bach2 is associated with Batf family transcription factors and inhibits AP-1 activity. The Bach2–Batf complex binds to AP-1 motifs within the regulatory regions of the Th2 cytokine gene loci and inhibits Th2 cytokine production. The increased production of IL-4 in *Bach2*-deficient naive CD4 T cells induces *Batf* and *Irf4*, and suppressed Bach2 expression. Furthermore, the Bach–Batf complex directly binds to the *Batf* and *Batf3* gene loci, and inhibits transcription. Therefore, Batf and Batf expression is augmented in *Bach2*-deficient CD4 T cells both in an IL-4-dependent and an IL-4-independent manner. The increased Batf and Irf4 form an active complex and further augments *Il4* expression. These findings reveal that IL-4 and the Batf /Irf4 form a positive feedback amplification loop to induce Th2 cell differentiation, and the Bach2–Batf complex is required to prevent the excessive induction of the Th2 response.

## Results

### Airway inflammation in T cell-specific *Bach2* KO mice

**Figure 1:**
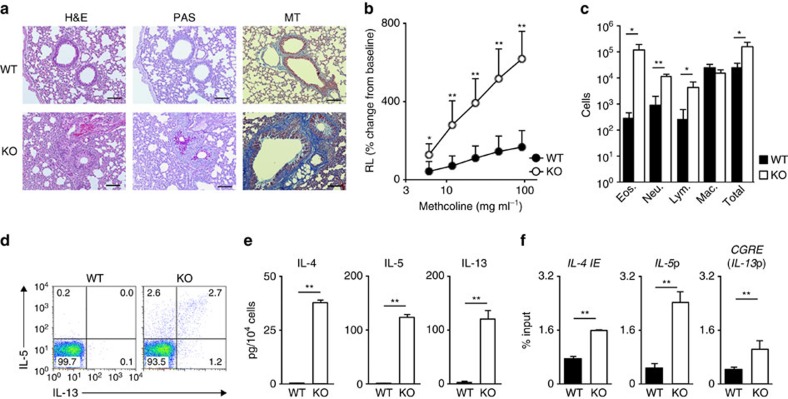
Spontaneous development of allergic airway inflammation in mice with T cell-specific *Bach2* deficiency. (**a**) Microscopic appearance of the lungs of wild-type and *Bach2*^fl/fl^ x CD4-Cre TG (*Bach2* KO) mice (*n*=5 per group), fixed and stained with haematoxylin and eosin (H&E; left panel), periodic acid-Schiff (PAS) reagent (middle panel) and Masson's trichrome (MT; right panel). Original magnification × 200 (Scale bars, 100 μm). (**b**) Airway resistance of the lungs of the wild-type or *Bach2* KO mice (mean±s.d., *n*=5 per group). *P<0.05 and **P<0.01 (ANOVA and Bonferroni's test). (**c**) Quantification of eosinophils, neutrophils, lymphocytes, macrophages and total cells in the BAL fluid of the wild-type and *Bach2* KO mice (mean±s.d., *n*=8 per group). *P<0.05 and **P<0.01 (Student's *t*-test). (**d**) The results of the intracellular flow cytometry analysis of IL-5 and IL-13 in lung CD4 T cells stimulated with 4-beta-phorbol 12-myristate acetate (PMA) plus ionomycin for 4 h. The numbers of cells are indicated in each quadrant. The data are representative of three-independent experiments with similar results. (**e**) The results of the ELISA for cytokines in the supernatants derived from the lung CD4 T cells stimulated with an immobilized anti-TCR-β mAb and an anti-CD28 mAb for 16 h (mean±s.d., *n*=3 per group). **P<0.01 (Student's *t*-test). (**f**) The levels of histone H3K27 acetylation at the Th2 cytokine gene loci in the lung CD4 T cells were determined using a ChIP-qPCR assay; the results are presented relative to those of input DNA with the s.d. **P<0.01 (Student's *t*-test) (mean±s.d., *n*=5).

In order to determine the intrinsic role of Bach2 in T cells, we crossed *Bach2*^flox/flox^ mice with *CD4-Cre* transgenic (TG) mice. A significant increase in mononuclear cells infiltrating the peribronchiolar regions of the lungs was observed in the *Bach2*^flox/flox^ CD4-Cre (*Bach*2-deficient) mice (Fig. 1a, left panel). The bronchioles of the *Bach*2-deficient mice showed mucus hyperproduction and goblet cell metaplasia, as assessed using staining with periodic acid-Schiff reagent (Fig. 1a, middle panel). The *Bach*2-deficient mice also exhibited an increased expression of *Clca3* messenger RNA (mRNA) and *Muc2* mRNA in the lungs versus the control CD4-Cre (WT) mice (Supplementary Fig. 1a). Moreover, pulmonary fibrosis was detected in the lungs of *Bach*2-deficient mice on Masson's trichrome staining (Fig. 1a, right panel), and methacholine-induced airway hyperresponsiveness was induced in the *Bach*2-deficient mice (Fig. 1b).

We observed increased infiltration of inflammatory cells, including eosinophils, neutrophils and lymphocytes, in the bronchoalveolar lavage (BAL) fluid of the *Bach*2-deficient mice compared with the control WT mice (Fig. 1c and Supplementary Fig. 1b). In addition, the numbers of IL-5- and IL-13-producing CD4 T cells were significantly increased in the lungs of *Bach*2-deficient mice compared with the control WT mice (Fig. 1d and Supplementary Fig. 1c). An increased production of Th2 cytokines (IL-4, IL-5 and IL-13) was also detected in lung CD4 T cells in the *Bach*2-deficient mice (Fig. 1e). Furthermore, the histone H3K27 acetylation levels at the Th2 cytokine gene loci were higher in the lung CD4 T cells of the *Bach*2-deficient mice than in the control WT mice (Fig. 1f). These results indicate that the *Bach*2-deficient mice spontaneously develop lung-specific Th2-type allergic inflammation.

Although the dysregulated development of Foxp3-positive CD4 T cells in *Bach2* null mice has previously been reported^20^,^29^, we detected no clear signs of inflammation in other organs (for example, the stomach, small and large intestines, liver, pancreas or kidneys) in the 8- to 12-week old T cell-specific *Bach*2-deficient mice (Supplementary Fig. 1d). The number of the Foxp3-positive CD4 T cells in the lungs the T cell-specific *Bach2*-deficient mice was equivalent to that in the WT control mice (Supplementary Fig. 2a). The number of Foxp3-positive CD4 T cells in the thymus of the T cell-specific *Bach*2-deficient mice was reduced to half that in the thymus of the WT control mice (Supplementary Fig. 2b). However, the numbers of Foxp3-positive CD4 T cells in other organs, such as the spleen, the mesenteric lymph nodes, Peyer's patch and the liver, were within in the normal ranges in the T cell-specific *Bach*2-deficient mice (Supplementary Fig. 2b).

### Stat6-dependent enhancement of Th2 response in *Bach2* KO mice

**Figure 2:**
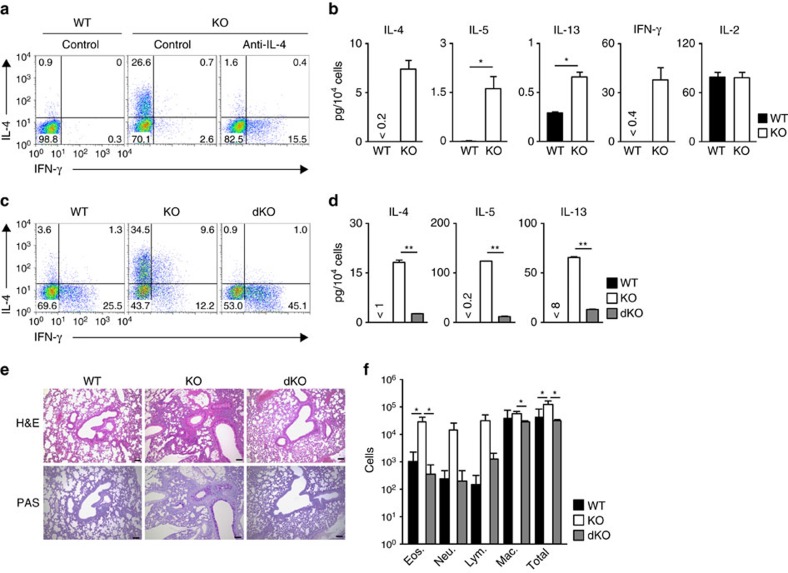
The IL-4/Stat6-dependent augmentation of the Th2-type immune response in *Bach2*-deficient mice. (**a**) The results of the intracellular flow cytometry analysis of IL-4/IFN-γ in the wild-type (WT) and *Bach2*-deficient naive CD4 T cells cultured under IL-2 conditions in the absence (left) or presence (right) of a neutralizing mAb against IL-4. The numbers of cells are indicated in each quadrant. The data are representative of three-independent experiments with similar results. (**b**) The results of the ELISA for cytokines in the supernatants of the WT and Bach2 KO naive CD4 T cells stimulated with an immobilized anti-TCR-β mAb and an anti-CD28 mAb for 16 h. *P<0.05 and **P<0.01 (Student's *t*-test) (mean±s.d., *n*=3 per group). The data are representative of at least three-independent experiments with similar results. (**c**) The results of the intracellular flow cytometry analysis of IL-4/IFN-γ in the WT, *Bach2*-deficient (KO) and *Bach2*/*Stat6* double-deficient (dKO) naive CD4 T cells cultured under IL-2 conditions. The numbers of cells are indicated in each quadrant. The data are representative of three-independent experiments with similar results. (**d**) The results of the ELISA for cytokines in the supernatants derived from wild-type (WT), *Bach2*-deficient (KO) and *Bach2*/*Stat6* double-deficient (dKO) lung CD4 T cells (mean±s.d., *n*=3 per group) stimulated with an immobilized anti-TCR-β mAb and an anti-CD28 mAb for 16 h. **P<0.01 (Student's *t*-test). (**e**) Microscopic appearance of the lungs of wild-type (WT), *Bach2*-deficient (KO) and *Bach2*/*Stat6* double-deficient (dKO) mice (mean±s.d., *n*=5 per group), fixed and stained with haematoxylin and eosin (H&E; upper panel) or periodic acid-Schiff (PAS) reagent (lower panel). Original magnification × 200 (Scale bars, 100 μm). (**f**) Quantification of eosinophils, neutrophils, lymphocytes, macrophages and total cells in the BAL fluid of the wild-type (WT), *Bach2*-deficient (KO) and *Bach2*/*Stat6* double-deficient (dKO) mice (mean±s.d., *n*=5 per group). *P<0.05 (Student's *t*-test).

To investigate the role of Bach2 in the differentiation of helper T (Th) cell subsets, we isolated *Bach*2-deficient naive CD4 T cells and cultured them under IL-2 conditions for five days in the presence or absence of a neutralizing monoclonal antibody (mAb) against IL-4. *Bach*2-deficient naive CD4 T cells cultured in the absence of a neutralizing mAb against IL-4 produced greater amounts of Th2 cytokines (IL-4, IL-5 and IL-13) than those derived from WT naive CD4 T cells (Fig. 2a and Supplementary Fig. 3a). The induction of Th2 cells in the *Bach2*-deficient naive CD4 T cells cultured under IL-2 conditions was blocked by the neutralization of IL-4 (Fig. 2a and Supplementary Fig. 3a). The expression of Gata3 (Supplementary Fig. 3b) and binding of Gata3 to the regulatory region of the Th2 cytokine gene locus such as Th2 LCR (Rad50 hypersensitive site 6; RHS6), *Il4* intron enhancer (*Il4* IE) and CGRE (Supplementary Fig. 3c) were increased in the *Bach*2-deficient CD4 T cells cultured under IL-2 conditions compared with the control WT CD4 T cells. The induction of Gata3 in the *Bach2*-deficient naive CD4 T cells cultured under IL-2 conditions was also IL-4-dependent (Supplementary Fig. 3b). The generation of IFN-γ-producing cells from the *Bach*2-deficient naive CD4 T cells was higher than that observed in the control WT CD4 T cells under IL-4 neutralizing conditions (Fig. 2a and Supplementary Fig. 3a). The induction of Foxp3-positive CD4 T cells was impaired in *Bach2*-deficient naive CD4 T cells under iTreg conditions (Supplementary Fig. 3d). We found that *Bach2*-deficient naive CD4 T cells produced significant amount of IL-4, IL-5, IL-13 and IFN-γ in response to T-cell antigen receptor (TCR) stimulation, whereas the production of IL-2 was comparable to the WT naive CD4 T cells (Fig. 2b). In addition, the sustained expression of *Il4* mRNA was detected in TCR-stimulated *Bach2*-deficient naive CD4 T cells (Supplementary Fig. 3e). We sorted the PD-1^high^ CXCR5^high^ fraction in the Peyer's patch of *Bach2*-deficient mice as a source of *in vivo* generated Tfh cells and then assessed the TCR-mediated induction of *Il4* mRNA expression. The expression of *Il4* mRNA in *Bach2*-deficient Tfh cells was significantly higher than that in control WT Tfh cells (Supplementary Fig. 3f, left panel). In contrast, the expression of *Il21* in *Bach2*-deficient Tfh cells was equivalent to that in the WT Tfh cells (Supplementary Fig. 3f, right panel). These results suggest that Bach2 has a critical role in regulating helper Th2 cell differentiation and/or Th2 cytokine production.

The increased generation of Th2 cells in *Bach2*-dficient CD4 T cells was also significantly reduced by *Stat6* deficiency (Fig. 2c and Supplementary Fig. 4a). In contrast, the generation of IFN-γ-producing cells was enhanced in *Bach2*-deficient CD4 T cells in the absence of the Stat6 (Fig. 2c). The increased production of Th2 cytokines in the lung CD4 T cells of *Bach*2-deficient mice was also reduced in *Bach2*/*Stat6* double-deficient mice (Fig. 2d), whereas the enhanced production of Th2 cytokines in naive *Bach2*-deficient splenic CD4 T cells was not normalized in *Bach2*/*Stat6* double-deficient naive CD4 T cells (Supplementary Fig. 4b). Although the reduced generation of Foxp3-positive cells in *Bach2*-deficient naive CD4 T cells was not restored by the deletion of *Stat6* (Supplementary Fig. 4c), the increased infiltration of mononuclear cells into the peribronchiolar regions of the lungs, and the mucus hyperproduction and goblet cell metaplasia observed in *Bach2*-deficient mice were improved in *Bach2*/*Stat6* double-deficient mice (Fig. 2e). The infiltration of eosinophils, neutrophils and lymphocytes in the BAL fluid of the *Bach*2-deficient mice was also significantly reduced by *Stat6*-deficiency (Fig. 2f and Supplementary Fig. 4d). The increased production of IL-17A and IL-17F was observed in the *Bach2*-deficient lung CD4 T cells, and the level was marginally affected by *Stat6* deletion (Supplementary Fig. 4e). These results clearly demonstrate that the spontaneously developed lung inflammation in T cell-specific *Bach2*-deficient mice is a Th2-type immune response.

### Decreased Th2 cell differentiation in *Bach2* TG CD4 T cells

**Figure 3:**
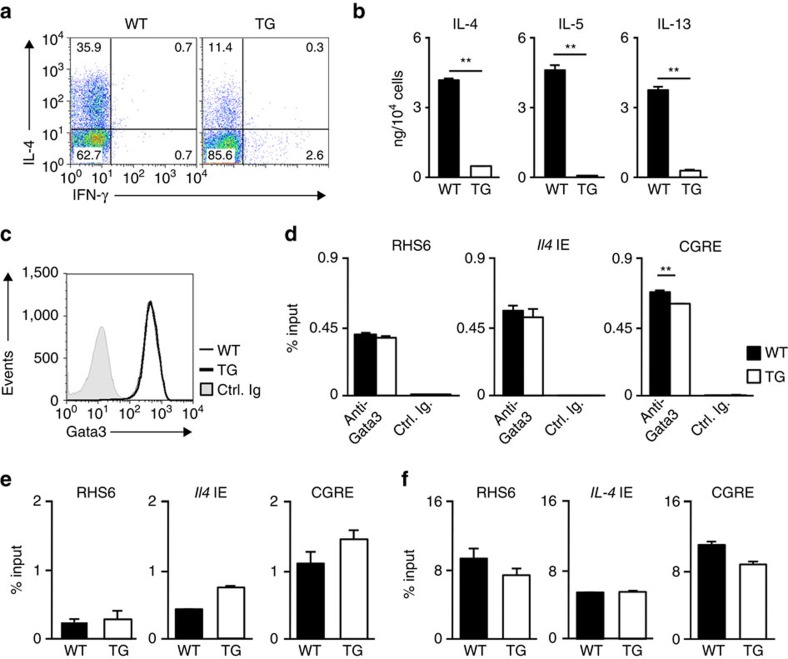
Decreased Th2 cell differentiation and Th2 cytokine production in *Bach2*-TG naive CD4 T cells. (**a**) The results of the intracellular flow cytometry analysis of IL-4/IFN-γ in the wild-type (WT) and *Bach2*-TG naive CD4 T cells cultured under Th2 conditions. The numbers of cells are indicated in each quadrant. The data are representative of at least three-independent experiments with similar results. (**b**) The results of the ELISA for cytokines in the supernatants of the cells in (**a**) stimulated with an immobilized anti-TCR-β mAb for 16 h. **P<0.01 (Student's *t*-test; mean±s.d., *n*=3). (**c**) The results of the flow cytometry analysis of Gata3 in WT or *Bach2*-deficient naive CD4 T cells cultured under Th2 conditions for 2 days. The data are representative of at least three-independent experiments with similar results. (**d**) The results of the ChIP assay of the binding of Gata3 to the *RHS6* within the LCR of the Th2 cytokine gene locus, the *Il4* IE and the CGRE in naive CD4 T cells cultured under Th2 conditions for 2 days; the results are presented relative to those of input DNA with the s.d. **P<0.01 (Student's *t*-test; *n*=3). (**e**,**f**) The levels of histone H3K4 tri-methylation (**e**) and H3K27 acetylation (**f**) at the Th2 cytokine gene loci in naive CD4 T cells cultured under Th2 condition for 2 days were determined using a ChIP-qPCR assay; the results are presented relative to those of input DNA with the s.d. **P<0.01 (Student's *t*-test; *n*=5).

To confirm the Treg-independent regulation of Th2-type immune response by Bach2, we used *Bach2* TG (Rosa26-Stop cassette^flox/flox^-*Bach2* TG mice crossed with *CD4-Cre* TG) mice (Supplementary Fig. 5a). The *Bach2* mRNA expression was ∼2.5-fold higher in the developing *Bach2* TG CD4 T cells than in the cells from WT mice (Supplementary Fig. 5b). The development of Foxp3-positive CD4 T cells was not changed in *Bach2* TG mice *in vivo* (Supplementary Fig. 5c) or *in vitro* (Supplementary Fig. 5d). There was less generation of IL-4-producing *Bach2* TG CD4 T cells than of IL-4-producing WT cells under Th2 conditions (Fig. 3a). We confirmed the decreased production of IL-4, IL-5 and IL-13 by the *Bach2* TG CD4 T cells cultured under Th2 conditions compared with the WT cells using an enzyme-linked immunosorbent assay (ELISA; Fig. 3b). Furthermore, the ovalbumin (OVA)-induced eosinophilic infiltration in the BAL fluid was attenuated in T cell-specific *Bach2* TG mice (Supplementary Fig. 5e,f). These results suggest that Bach2 can suppress Th2 cytokine production and/or Th2 cell differentiation independent of Treg-mediated regulation. Although the production of Th2 cytokine was impaired, the expression of Gata3 protein (Fig. 3c) and the binding of Gata3 to the regulatory regions of the Th2 cytokine gene loci in the *Bach2* TG-developing Th2 cells (cultured under Th2 conditions for 2 days) were comparable to those in the WT Th2 cells (Fig. 3d). The histone H3K4 tri-methylation (Fig. 3e) and H3K27 acetylation (Fig. 3f) levels at the Th2 cytokine gene loci were not decreased in the *Bach*2-deficient developing Th2 cells. These results suggest that Bach2 can suppress Th2 cytokine production without inhibiting the chromatin remodelling of the Th2 cytokine gene loci.

### Bach2 binds to the AP-1 consensus motif

**Figure 4:**
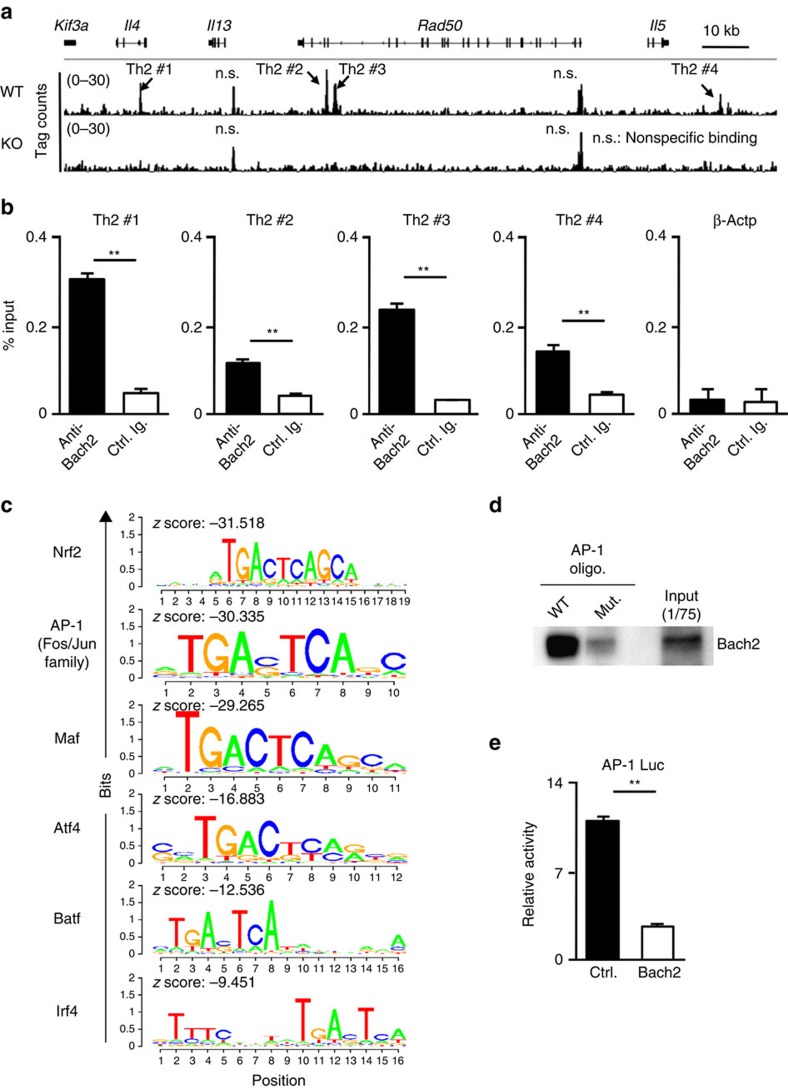
Bach2 binds to the AP-1 motif containing regulatory regions of Th2 cytokine gene loci. (**a**) Global patterns of Bach2 binding at the Th2 cytokine gene loci in the WT and *Bach2*-deficient naive cells cultured under neutral conditions for 5 days were determined using a ChIP-seq analysis. (**b**) The results of the ChIP assay with a quantitative PCR analysis of Bach2 binding in the WT naive CD4 T cells cultured under IL-2 conditions for 2 days; the results are presented relative to those of input DNA with the s.d. **P<0.01 (Student's *t*-test; *n*=3). (**c**) Enriched Bach2-binding motifs in the naive CD4 T cells cultured under neutral conditions for 5 days. (**d**) The results of the pull-down assay of the binding of Bach2 to wild-type (WT) or mutant (Mut.) AP-1 consensus oligonucleotides in the effector CD4 T cell lysates. The data are representative of at least three-independent experiments with similar results. (**e**) The luciferase activity in 293 T cells transfected with an empty vector or Bach2, as well as a firefly luciferase reporter for AP-1, was unstimulated or stimulated with the phorbol ester 4-beta-phorbol 12-myristate acetate (PMA) (30 ng ml^−1^) for 16 h; the results are presented relative to the *Renilla* luciferase activity with the s.d. **P<0.01 (Student's *t*-test; *n*=3).

In order to investigate the regulatory mechanisms by which Bach2 controls Th2 cytokine production and/or Th2 cell differentiation, we performed a ChIP-seq analysis with an anti-Bach2 antibody to determine Bach2-binding sites. The enrichment of the Bach2-binding motifs in WT effector CD4 T cells cultured under neutral conditions was significantly increased relative to the background frequency estimated based on a *Bach2*-deficient effector CD4 T cell ChIP sample. We found that Bach2 binds to the *Il4* intron enhancer (*Il4* IE) (Th2 #1), the RHS6 located within the Th2 LCR (Th2 #2 and Th2 #3) and the 3′ downstream region of the *Il5* gene (Th2 #4) (Fig. 4a). The binding of Bach2 was confirmed using a ChIP-quantitative PCR (qPCR) analysis (Fig. 4b). The major enriched motifs for Bach2 binding were the Nrf2, AP-1, Maf, Atf4, Batf and Irf4-binding sites (Fig. 4c). We found that enriched Bach2-binding sites in CD4 T cells were binding motifs for leucine zipper family transcription factors and contain the AP-1 consensus DNA sequence (TGA(G/C)TCA) (Fig. 4c). We next performed an oligonucleotide pull-down analysis to examine whether Bach2 binds to the AP-1 consensus motif. An AP-1 consensus oligonucleotide precipitated Bach2 from activated CD4 T cell lysates, while an AP-1 mutant oligonucleotide failed to precipitate Bach2 (Fig. 4d). Furthermore, Bach2 repressed the 4-beta-phorbol 12-myristate acetate-induced AP-1 luciferase activity in 293 T cells (Fig. 4e). These results indicate that Bach2 binds to AP-1 motifs and inhibits AP-1-dependent gene activation.

### Bach2 interacts with Batf family transcription factors

**Figure 5:**
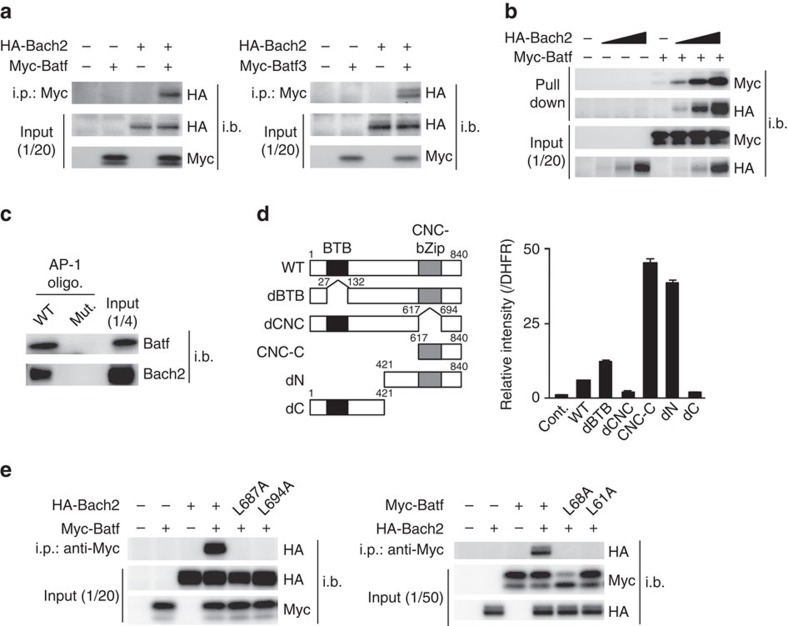
The association of Bach2 with Batf family proteins. (**a**) Immunoprecipitation (i.p.) and immunoblot (i.b.) analyses of the association of Bach2 and Batf in 293 T cell lysates left untransfected (−) or transfected (+) to express Myc-tagged Batf (Myc-Batf) and/or HA-tagged Bach2 (HA-Bach2; left) or to express Myc-tagged Batf (Myc-Batf3) and/or HA-tagged Bach2 (HA-Bach2; right); below (Input), a parallel analysis of the total cell lysates (without i.p.). The data are representative of three-independent experiments with similar results. (**b**) A pull-down assay of the binding of the Batf/Bach2 complex to an AP-1 consensus oligonucleotide in 293 T cell lysates transfected to express Myc-Batf and/or HA-Bach2 (Input (below), i.b. analysis of whole-cell lysates without precipitation). Wedges indicate 3-fold ‘titration' of the input lysates. The data are representative of independent experiments with similar results. (**c**) The results of the pull-down assay of the binding of Bach2–Batf complex to wild-type (WT) or mutant (Mut.) AP-1 consensus oligonucleotides in the effector CD4 T cell lysates. The data are representative of three-independent experiments with similar results. (**d**) A schematic representation of the Bach2 mutants (left). The results of the AlphaScreen to detect the interaction of Batf with the Bach2 mutants (right). The relative intensities to dihydrofolate reductase (DHFR) binding are presented as the averages of three-independent experiments. (**e**) i.p. and i.b. analyses of the association of the wild-type Batf with Bach2 point mutants (L687A and L694A) (left) or wild-type Bach2 with Batf point mutants (L68A and L61A) (right l) in 293 T cell lysates. The cell lysates were prepared as described in (**a**). The data are representative of three-independent experiments with similar results.

In order to understand the molecular mechanisms by which Bach2 binds to the AP-1 motif and suppresses AP-1 function, we screened Bach2 interaction transcriptional modulator(s) using an amplified luminescent proximity homogenous assay (AlphaScreen) with cell-free technology for protein synthesis as previously described^30^,^31^. Sixty-five transcriptional modulators were screened, and Batf and Batf3 were identified to be potential candidates for Bach2 interacting transcription factors (Supplementary Fig. 6). We confirmed that Batf was immunoprecipitated with Bach2 in mixtures of 293 T cells transfected to express Myc-tagged Batf or HA-tagged Bach2 (Fig. 5a, left). Batf3 was also immunoprecipitated with Bach2 in mixtures of 293 T cells (Fig. 5a, right). Next, we performed an oligonucleotide precipitation assay to confirm the DNA-binding activity of the Bach2–Batf complex. Binding of the Bach2–Batf complex to the AP-1 consensus oligonucleotide was detected only when both proteins were in the lysates, and the level of Batf binding was increased in a Bach2 dose-dependent manner (Fig. 5b). Bach2 and Batf were not precipitated when both proteins were present independently in the lysates (Fig. 5b). The binding of Bach2–Batf complex to the AP-1 consensus oligonucleotide was also detected in activated CD4 T cell lysates (Fig. 5c). To identify the Bach2 protein regions, which are required for the association with Batf, we generated several Bach2 deletion mutants (Fig. 5d left) and assessed the interaction with Batf using AlphaScreen as in Supplementary Fig. 6. The Batf dCNC and dC mutants failed to associate with Batf, while the CNC-C and dN mutants strongly interacted with Batf compared with the WT Bach2 protein (Fig. 5d right), indicating that the CNC-bZip region of Bach2 is responsible for the interaction with Batf. To confirm the important role of bZip regions, we introduced a point mutation into the leucine zipper region of Bach2 (L687A and L694A) and Batf (L68A and L61A), and assessed the interactions. As expected, the interaction of Bach2 and Batf was completely abrogated by the point mutation in the leucine zipper region of each protein (Fig. 5e). The results clearly demonstrated that the leucine zipper of both proteins is responsible for the interaction.

### Bach2–Batf complex antagonizes the Batf–Irf4 complex

**Figure 6:**
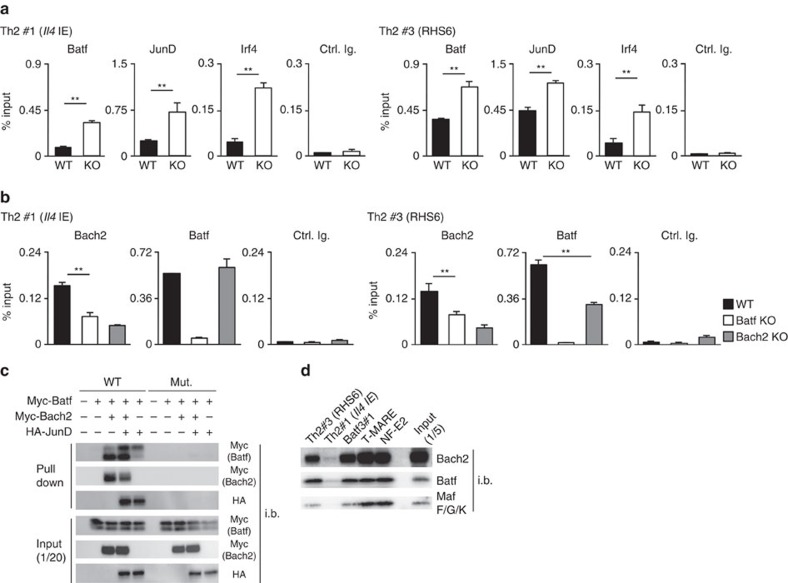
The Bach2/Batf complex interferes with the recruitment of the Batf/JunD/Irf4 active complex. (**a**) The results of the ChIP assay of Batf, JunD or Irf4 binding at Th2#1 (*Il4* IE) and Th2#3 (RHS6 region) using a quantitative PCR (qPCR) analysis with wild-type (WT) and *Bach2*-deficient CD4 T cells cultured under IL-2 conditions for 48 h; the results are presented relative to those of input DNA with the s.d. *P<0.05 and **P<0.01 (Student's *t*-test; *n*=3). The data are representative of at least three-independent experiments with similar results. (**b**) The results of the ChIP assay of Bach2 or Batf binding at Th2#1 (*Il4* IE) and Th2#3 (RHS6) using a qPCR analysis with WT, *Batf*-deficient and *Bach2*-deficient CD4 T cells cultured under IL-2 conditions for 48 h; the results are presented relative to those of input DNA with the s.d. *P<0.05 and **P<0.01 (Student's *t*-test; *n*=3). The data are representative of at least three-independent experiments with similar results. (**c**) A pull-down assay of binding of the Batf/Bach2 complex and Batf/JunD complex to a Th2#3 oligonucleotide (WT) or AP-1 motif-mutated Th2#3 oligonucleotide (Mut.) in 293 T cell lysates left untransfected (−) or transfected (+) to express Myc-Batf, Myc-Bach2 and/or HA-tagged JunD (HA-JunD); below (Input), a parallel analysis of total cell lysates (without immunoprecipitation). The data are representative of three-independent experiments with similar results. (**d**) The results of the pull-down assay of the binding of Bach2–Batf complex to the RHS6 and Batf3#1 oligonucleotide in the Th2 cell lysates. The data are representative of three-independent experiments with similar results.

We next examined the effects of *Bach2*-deficiency on binding of the Batf–Irf4 complex using a ChIP assay. The binding of Batf, JunD and Irf4 to the *Il4* IE (Th2 #1) and RHS6 (Th2 #3) regions was significantly increased in the *Bach2*-deficient activated CD4 T cells as compared with that observed in the WT CD4 T cells (Fig. 6a). Additionally, the levels of histone H3K27 acetylation at the *Il4* IE (Th2#1) and RHS6 (Th2 #3) regions were elevated in the *Bach2*-deficient naive CD4 T cells cultured under both neutral (in the presence of an anti-IL-4 mAb) and Th2 conditions (Supplementary Fig. 7). We confirmed that the binding of Bach2 to the *Il4* IE (Th2 #1) and RHS6 (Th2 #3) (Fig. 6b) regions were significantly decreased in the *Batf*-deficient CD4 T cells. Furthermore, the binding level of Batf at the RHS6 (Th2 #3) region was reduced in the *Bach2*-deficient CD4 T cells (Fig. 6b).

We performed an oligonucleotide precipitation assay to confirm the DNA-binding activity of the Batf–Bach2 complex to the RHS6 oligonucleotide (Th2#3), which contains an AP-1-binding site. When we used 293 T cell lysates that transfected with Myc-tagged Batf, Myc-tagged Bach2 or HA-tagged JunD, two distinct mobility forms of the Myc-tagged Batf protein were precipitated (Fig. 6c, upper). We found that Bach2 preferentially associated with a faster mobility form of Batf, whereas JunD interacted with a slower mobility form of Batf (Fig. 6c, upper). These data suggest that the post-translational modification of Batf may influence the ratio of the repressive-type (Bach2–Batf) and activate-type (Batf–JunD–Irf4) Batf complex. The binding of Bach2 to the WT RHS6 oligonucleotide was moderately reduced in the presence of JunD (comparison of lane 3 and lane 4, Fig. 6c second panel). The RHS6 mutant oligonucleotide, which has a mutation at the AP-1-binding site, failed to precipitate either complex (Fig. 6c, upper), indicating that Bach2–Batf complex recognized the AP-1 motif in the RHS6 oligonucleotide. Finally, we assessed the binding of Bach2–Batf complex to the Bach2 binding sites identified by ChIP-sequencing using Th2 cell lysates. Bach2 and Batf were efficiently precipitated by the RHS6 and Batf3#3 (the Bach2-binding site of the *Batf3* gene locus) oligonucleotides, while the binding of Bach2 and Batf to the *Il4* IE oligonucleotide was relatively weak (Fig. 6d, upper and middle). The binding of Bach2 and Batf to the *Il4* IE region was clearly detected by a ChIP-qPCR assay (Figs 4b and 6a). It is likely that a Th2-specific high order chromatin structure is required for the binding of Bach2 and Batf at the *Il4* IE region. The relatively weak binding of the small Maf protein(s) to the RHS6 and Batf3#3 oligonucleotides was detected (Fig. 6d, lower). The precipitation of Bach2 and Batf by the T-MARE and NF-E2 consensus oligonucleotides (Fig. 6d) suggested that the Bach2–Batf complex could bind to the Nrf2-binding sites in T cells. From these results, we concluded that the Bach2–Batf complex primarily binds to the RHS6 region and that it negatively regulates Th2 cytokine expression via the modulation of Batf–JunD–Irf4 binding.

### Bach2–Batf complex regulates the *Batf* and *Batf3* expression

**Figure 7:**
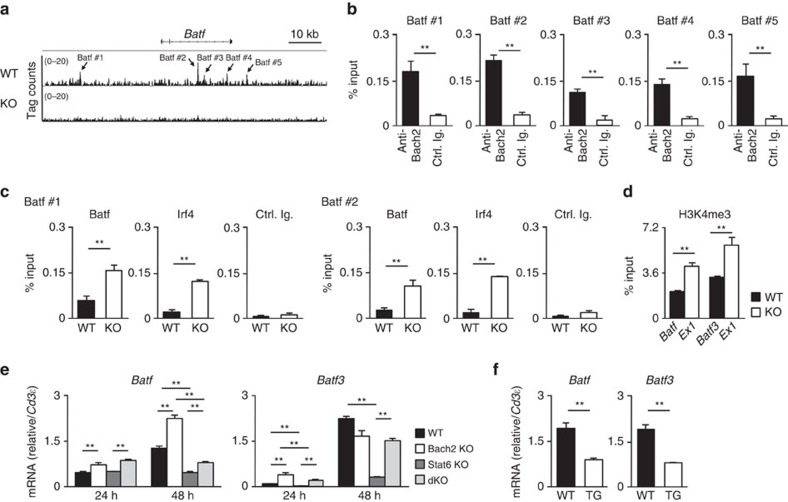
Bach2 inhibits the formation of a positive feedback loop to induce Th2 cell development. (**a**) Global patterns of Bach2 binding to the *Batf* gene locus in the wild-type (WT) and *Bach2*-deficient naive cells cultured under neutral conditions for 5 days were determined using a ChIP-seq analysis. (**b**) Results of the ChIP assay with a quantitative PCR (qPCR) analysis of Bach2 binding to the *Batf* gene locus in the WT naive CD4 T cells cultured under IL-2 conditions for 48 h; the results are presented relative to those of input DNA with the s.d. **P<0.01 (Student's *t*-test; *n*=3). (**c**) Results of the ChIP assay with a qPCR analysis of Batf or Irf4 binding at the Batf #1 and Batf #2 regions in the WT and *Bach2*-deficient CD4 T cells cultured under neutral conditions for 48 h; the results are presented relative to those of input DNA with the s.d. *P<0.05 and **P<0.01 (Student's *t*-test; *n*=3). The data are representative of three-independent experiments with similar results. (**d**) The levels of histone H3K4 tri-methylation at the transcription start sites of the *Batf* and Batf3 gene loci in the WT or *Bach2*-deficient naive CD4 T cells cultured under IL-2 conditions for 48 h were determined using a ChIP-qPCR assay; the results are presented relative to those of input DNA with the s.d. **P<0.01 (Student's *t*-test; *n*=3). (**e**) Results of the quantitative reverse transcription (RT)–PCR analysis of *Batf* and *Batf3* mRNA in the WT, *Bach2*-, *Stat6*- or *Bach2*/*Stat6* double-deficient naive CD4 T cells stimulated with an anti-TCR-β mAb plus an anti-CD28 mAb in the presence of IL-2 for the indicated hours. The results are presented relative to the mRNA expression of *Cd3*ɛ, with the s.d. **P<0.01 (Student's *t*-test; *n*=3). (**f**) Results of the quantitative RT–PCR analysis of *Bach2* and *Batf3* mRNA in the WT or *Bach2* TG naive CD4 T cells cultured under Th2 conditions for 48 h. The results are presented relative to the mRNA expression of *Cd3*ɛ, with the s.d. **P<0.01 (Student's *t*-test; *n*=3).

We detected Bach2 binding to the *Batf* and *Batf3* gene loci using ChIP-sequencing (Fig. 7a and Supplementary Fig. 8a). Binding of Bach2 to the *Batf* and *Batf3* gene loci was confirmed using a ChIP assay with qPCR (Fig. 7b and Supplementary Fig. 8b). Furthermore, binding of Batf and Irf4 to the Bach2-binding sites within the *Batf* and *Batf3* gene loci was significantly increased in the *Bach2*-deficient CD4 T cells cultured under neutral conditions (Fig. 7c and Supplementary Fig. 8c), and the histone H3K4 tri-methylation levels at the transcription starting sites (*Batf* exon1 and *Batf3* exon1 regions) of *Batf* and *Batf3* were elevated in the *Bach2*-deficient effector CD4 T cells (Fig. 7d). As previously reported^32^, we observed IL-4/Stat6-dependent maintenance of *Batf*, *Batf3* and *Irf4* expression (Supplementary Fig. 9). However, the sustained mRNA expression of *Batf* and *Batf3* in the TCR-stimulated *Bach2*-deficient naive CD4 T cells showed an only partial reduction in *Bach2*/*Stat6* double deficient developing Th2 cells (Fig. 7e). Moreover, the decreased expressions of *Batf* and *Batf3* mRNA was observed in the *Bach2* TG CD4 T cells cultured under Th2 conditions (in the presence of IL-4) (Fig. 7f) suggesting that Bach2 can directly suppress the expression of *Batf* and *Batf3*. Interestingly, IL-4/Stat6-signalling inhibited *Bach2* expression (Supplementary Fig. 9). These results indicate that the activation of the IL-4/Stat6 signalling pathway induces the sustained formation of the Batf (Batf3)–Irf4 complex. Thus, Bach2 regulates the expression of *Batf* and *Batf3* via two distinct pathways. First, Bach2 suppresses the IL-4/Stat6-dependent maintenance of *Batf* and *Batf3* expression through the inhibition of IL-4 production. Second, the Bach2–Batf complex binds directly to the *Batf* and *Batf3* gene loci and reduces transcription by interfering with the recruitment of the Batf–Irf4 complex.

### Batf deficiency normalizes Th2 response in Bach KO mice

**Figure 8:**
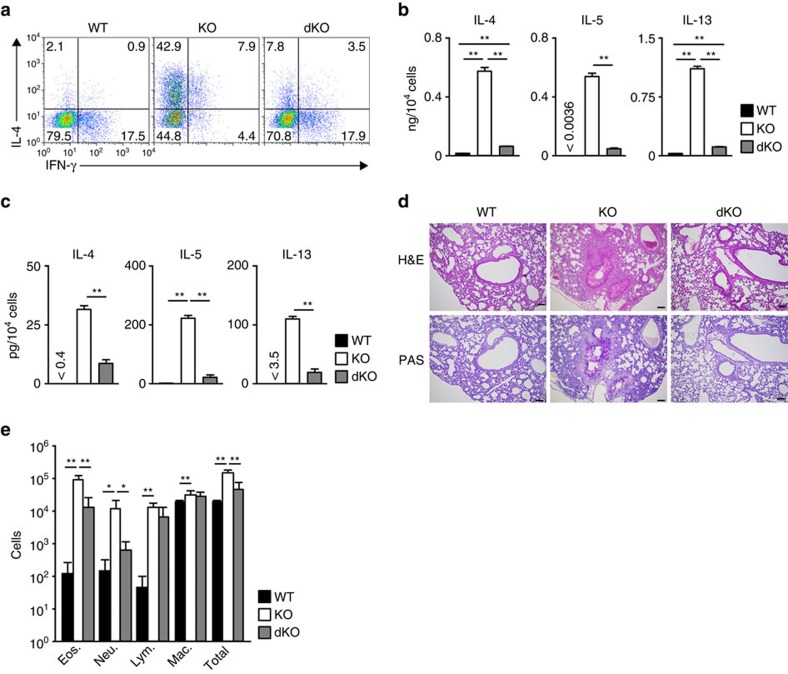
The augmented Th2-type immune response in the *Bach2*-deficient mice was normalized by T cell-specific *Batf* deletion. (**a**) The results of the intracellular flow cytometry analysis of IL-4/IFN-γ in the WT, *Bach2*-deficient (KO) and *Bach2*/*Batf* double-deficient (dKO) naive CD4 T cells cultured under IL-2 conditions. The numbers of cells are indicated in each quadrant. The data are representative of three-independent experiments with similar results. (**b**) Results of the ELISA for cytokines in the supernatants of the cells in (**a**) stimulated with an immobilized anti-TCR-β mAb for 16 h. **P<0.01 (Student's *t*-test; mean±s.d., *n*=3). (**c**) Results of the ELISA for cytokines in the supernatants derived from the lung CD4 T cells from indicated mice stimulated with an immobilized anti-TCR-β mAb and an anti-CD28 mAb for 16 h. **P<0.01 (Student's *t*-test; mean±s.d., *n*=4). (**d**) Microscopic appearance of the lungs of wild-type (WT), *Bach2*-deficient (KO) and *Bach2*/*Batf* double-deficient (dKO) mice (mean±s.d., *n*=5 per group), fixed and stained with haematoxylin and eosin (H&E; upper panel) or periodic acid-Schiff (PAS) reagent (lower panel). Original magnification × 200 (Scale bars, 100 μm). (**e**) Quantification of eosinophils, neutrophils, lymphocytes, macrophages and total cells in the BAL fluid of the wild-type (WT), *Bach2*-deficient (KO) and *Bach2*/*Batf* double-deficient (dKO) mice (mean±s.d., *n*=5 per group). *P<0.05 and **P<0.01 (Student's *t*-test).

Finally, we assessed whether the T cell-specific deletion of the *Batf* (Supplementary Fig. 10a) normalized the augmented Th2-type immune response in *Bach2*-deficient mice to confirm the involvement of Bach2-Batf interactions. The increased generation of IL-4-producing Th2 cells in the *Bach2*-deficient mice was significantly reduced by *Batf* deficiency (Fig. 8a). The increased Th2 cytokine production in *Bach2*-deficient CD4 T cells was also normalized by *Batf* deficiency (Fig. 8b). The reduced generation of Foxp3-positive CD4 T cells in *Bach2*-deficient mice was not restored by the deletion of *Batf in vitro* or *in vivo* (Supplementary Fig. 10b,c). The increased production of Th2 cytokines (IL-4, IL-5 and IL-13) in CD4 T cells from the lungs of *Bach*2-deficient mice was reduced by the deletion of *Batf* (Fig. 8c). The increased infiltration of mononuclear cells into the peribronchiolar regions of the lungs (Fig. 8d, upper), and mucus hyperproduction and goblet cell metaplasia (Fig. 8d, lower), which were observed in *Bach2*-deficient mice was improved by the deletion of *Batf*. The increased infiltration of eosinophils and neutrophils that was observed in the BAL fluid of the *Bach*2-deficient mice was also significantly reduced in the *Bach2*/*Batf* double-deficient mice (Fig. 8e and Supplementary Fig. 10d). Taken together, we conclude that IL-4 and Batf-Irf4 form a positive feedback loop to induce Th2 cell differentiation, and Bach2–Batf interactions interfere with this amplification loop (Supplementary Fig. 11).

## Discussion

The CNC gene family consists of two transcriptional repressors (Bach1 and Bach2) and four activators (NF-E2, Nrf1, Nrf2 and Nrf3; refs 11, 12). Bach2 interacts with small Maf proteins and acts as a genetic inhibitor of the gene expression directed by the 12-o-tetradecanoyl phorbol-13-acetate (TPA) response element, Maf recognition element and antioxidant response element^33^,^34^. Bach2-binding sites in effector CD4 T cells are enriched predominantly in the motifs for AP-1, Nrf/Maf (MARE: Maf response element), Atf4, Batf and Irf4. We found that the majority of enriched motifs contain the AP-1 consensus DNA sequence (TGA(G/C)TCA). Although Bach2 has a crucial role in T-cell-mediated immune responses^19^,^21^,^23^ , the role of small Maf proteins in T-cell-mediated immune responses has not yet been established. It was recently reported that Bach2 regulates CD8 T cell differentiation by controlling the access of AP-1 factors to enhancers. However, the molecular mechanism (s) by which Bach2 inhibits AP-1 access to enhancers is poorly understood^35^. In the present study, we identified Batf family proteins, Batf and Batf3 as binding partners of Bach2. Batf is a member of the AP-1 family of bZip transcription factors that negatively regulates AP-1-dependent transcription^25^,^26^. It is also well established that the interaction of Batf with Jun and Irf family proteins activates AP-1-dependent transcription. In the current study, we detected the increased binding of Irf4 and JunD to the Bach2 target genes loci in *Bach2*-deficient CD4 T cells. Taken together, these findings suggest the likelihood that Bach2 associates with Batf and suppresses AP-1-dependent gene activation by interfering with the recruitment of Irf4-containing active-type Batf complex.

We found that IL-4 and Batf interact in a positive feedback loop and Bach2–Batf interactions have a critical role in inhibiting this amplification loop. The LCR for the Th2 cytokine gene loci has been mapped to a region of ∼25-kb within the 3′ intronic regions of the *Rad50* genes^8^. DNA hypersensitivity analyses have revealed the presence of several evolutionally conserved hypersensitive sites, named RHS4–7 (ref. 8). The HS2, *Il4* IE region is crucial for the production of IL-4 by CD4 T cells^9^, and deletion of the *Il4* IE site result in the reduction of IL-4 production, but not that of IL-5 or IL-13, in Th2 cells. We found that Bach2 binds to the RHS6 and *Il4* IE regions by ChIP-sequencing, and the production of IL-4, IL-5 and IL-13 was dramatically increased in the *Bach2*-deficient effector CD4 T cells. The binding of the Batf/JunD/Irf4 complex at the RHS6 and *Il4* IE regions was detected by a ChIP-qPCR, and the levels were higher in the *Bach2*-deficient activated CD4 T cells than in the WT activated CD4 T cells. The Bach2–Batf complex was efficiently precipitated by the RHS6 (Th2#3) oligonucleotide. However, we only detected the weak binding of Bach2 and Batf to the DNA probe of the *Il4* IE (Th2#1) region by an oligonucleotide precipitation assay. It is likely that a Th2-specific high-order chromatin structure is required for the binding of Bach2–Batf complex at the *Il4* IE region. These results suggest that the Bach2–Batf complex binds to the AP-1 motif within the RHS6 and interferes with the recruitment of active-type AP-1 complexes such as the Batf/JunD/Irf4 complex. Hence, the recruitment of the Batf/JunD/Ifr4-containing AP-1 complex was increased in the *Bach2*-deficient CD4 T cells, and Th2 cytokine production was consequently augmented.

In addition to Th2 cytokines, we identified *Batf* and *Batf3* as direct targets of Bach2-mediated repression. The expression levels of *Batf* and *Batf3* were augmented in the *Bach2*-deficient activated CD4 T cells, and a significant increase in Irf4 binding at the Bach2-binding regions within the *Batf* and *Batf3* gene loci was detected. These results indicate that activation of the *Batf* and *Batf3* genes is also inhibited by Bach2–Batf interactions. Furthermore, we found that the activation of the IL-4/Stat6 signalling pathway augmented the TCR-mediated induction of *Batf*, *Batf3*, *Irf4* and suppressed the *Bach2* expression. Therefore, we propose the existence of a regulatory network among IL-4, the Bach2/Batf complex and the Batf/Irf4 complex that controls Th2 differentiation and Th2-type immune response.

The previous reports showed that the number of nTreg cells was significantly decreased in *Bach2*-null mice^20^. They also demonstrated the hematopoietic cell-intrinsic role of Bach2 in the development and/or survival of Foxp3-positive CD4 T cells by bone marrow chimera experiments. Although the number of Foxp3-positive CD4 T cells was not reduced in the peripheral organs including lungs of T cell-specific *Bach2*-deficient mice, T cell-specific *Bach2*-deficient mice spontaneously developed Th2-type lung inflammation. We demonstrated that *Stat6* or *Batf* gene depletion suppressed the spontaneous development of lung inflammation induced by T cell-specific *Bach2* deficiency. The deletion of the *Stat6* or *Batf* gene in *Bach2*-deficient naive CD4 T cells failed to restore the generation of Foxp3-positive iTreg cells *in vitro*. Furthermore, Th2 cell differentiation was inhibited in *Bach2* Tg naive CD4 T cells without the influence of iTreg cell development. These results clearly demonstrate that Bach2 regulates Th2 cell development and Th2-type lung inflammation, in part, through a Treg-independent mechanism(s).

We found that the number of IL-33 receptor α (IL-33rα-positive lung CD4 T cell cells that express *Il4*, *Il5* and *Il13* mRNA were increased in *Bach2*-deficient mice (Supplementary Fig. 12a,b). The increased cell surface expression of IL-33rα was marginal on the CD4 T cells in other organs. The characteristic feature of the IL-33rα-positive *Bach2*-deficient lung CD4 T cells was similar to that of the pathogenic Th2 cells, as was recently proposed by Endo and Nakayama *et al*^36^. Furthermore, we found that *Il33* mRNA was increased in the lung of *Bach2*-deficient mice (Supplementary Fig. 12c). The selective upregulation of IL-33rα expression in *Bach2*-deficient lung CD4 T cells and the enhanced expression of *Il33* in the lungs of *Bach2*-deficient mice may explain why the inflammatory incident solely occurs in the lungs.

A previous study of the comprehensive identification of bZip proteins did not detect the interaction of Bach2 with Batf^37^. The binding strength between MafK and Bach2 was ∼20times higher than that of the Batf–Bach2 interaction assessed by our AlphaScreen system. It is, therefore, likely that the authors of the previous study failed to detect the Bach2–Batf interactions due to low sensitivity of their assay system. However, the oligonucleotide pull-down assay revealed that the binding of Batf to the RHS6 was equivalent that of MafK. We detected two distinct mobility forms of Myc-tagged Batf and HA-tagged Bach2 proteins in the lysates obtained from transfected 293 T cells. Bach2 selectively precipitates with the faster-migrating form, whereas JunD preferentially associates with the slower-migrating form. It has been reported that Batf is phosphorylated on multiple serine and threonine residues and at least one tyrosine residue^38^. Furthermore, the regulation of Bach2 function and translocation by Bach2 was also recently reported^35^,^39^. It is likely that the posttranslational modifications of Batf and Bach2 control the strength of the Bach2–Batf interactions.

In conclusion, we herein demonstrated that Bach2 interacts with Batf family transcription factors and controls Th2 cell development via binding to the regulatory regions in the Th2 cytokine, *Batf* and *Batf3* gene loci. We demonstrate that IL-4 and the Batf/Irf4 complex form a positive feedback loop for Th2 cell differentiation, whereas the Bach2–Batf interactions inhibit the formation of this loop. Thus, T cell-specific *Bach2* deficiency in mice results in the accelerated a positive feedback loop and subsequent enhancement of the Th2-type immune response. Taken together, we propose a novel model for the IL-4 amplifier to induce chronic Th2-type inflammation. In addition, the data presented herein also implying the novel regulatory mechanism of T cell differentiation and function mediated by Bach2–Batf interactions.

## Methods

### Mice

Cre TG mice under the control of the *Cd4* promoter were purchased from The Jackson Laboratory. *Bach2*^flox/flox^ mice^16^, Rosa-stop-Flag-Bach2 knock in mice (conditional *Bach2* TG mice) and *Batf*^flox/flox^ mice were established by Dr Tomohiro Kurosaki (RIKEN and Osaka University) and C57BL/6 mice were purchased from Clea (Clea Japan, Inc., Tokyo, Japan). *Stat6*-deficient mice^40^ were kindly provided by Dr Shizuo Akira (Osaka University). Gene manipulated mice with C57BL/6 background were used in all experiments. Female mice were used in the *in vivo* experiments. Both male and female mice were used in the *in vitro* experiments. All mice were maintained under specific pathogen-free conditions and used at 8–12 weeks of age. We did not use blind methods. All experiments using mice received approval from the Kazusa DNA Research Institute and Ehime University Administrative Panel for Animal Care. All animal care was conducted in accordance with the guidelines of the Kazusa DNA Research Institute and Ehime University.

### Reagents

The antibodies used for intracellular and cell-surface staining were as follows: anti-IL-4-phycoerythrin mAb (11B11; BD Bioscience), anti-IFN-γ-fluorescein isothiocyanate isomer-1 (FITC) mAb (XMG1.2; BD Bioscience), anti-IL-5-allophycocyanin (APC) (TRFK5; eBioscience), anti-IL-13-phycoerythrin (eBio13A; eBioscience) and anti-Gata3-Alexa Fluor 647 (L50-823; BD Bioscience). The antibodies used for immunoblotting were as follows: anti-Gata3 mAb (cat#A300-105A; Santa Cruz Biotech) and anti-α-Tubulin mAb (cat#MS-581-P; NeoMarkers). The antibodies used for the ChIP assay were as follows: anti-Bach2 anti-serum (cat#SAB2100201; Thermo Scientific), anti-Gata3 pAb (cat#AF2605; R&D), ant-Batf mAb (D7C5; CST), anti-Irf4 mAb (D43H10; CST), anti-JunD pAb (329; Santa Cruz Biotech), anti-histone H3K4me3 pAb (cat#39159; Active Motif) and anti-histone H3K27ac pAb (cat#39133; Active Motif). The antibodies used for the immunoprecipitation were as follows: anti-Myc-Tag mAb (PL14; MBL, Japan) and anti-HA-Tag mAb (TANA2; MBL, Japan). All antibodies were diluted and used according to the manufacturer's instructions.

### CD4 T cells stimulation and differentiation *in vitro*

Naive CD4 T (CD44^low^CD62L^high^CD25^negative^) cells were prepared using a CD4^+^CD62L^+^ T cell isolation kit II (cat#130-093-227; Miltenyi Biotec). Naive CD4 T cells (1.5 × 10^6^) were stimulated with an immobilized anti-TCR-β mAb (3 μg ml^−1^, H57-597; BioLegend) and an anti-CD28 mAb (1 μg ml^−1^, 37.5; BioLegend) for 2 days under the conditions indicated. Next, the cells were transferred to a new plate and further cultured in the presence of cytokines. The cytokine conditions were as follows: IL-2 conditions, IL-2 (10 ng ml^−1^); Neutral (Thn) conditions, IL-2 (10 ng ml^−1^), anti-IL-4 mAb (5 μg ml^−1^, 11B11; BioLegend) and anti-IFN-γ mAb (5 μg ml^−1^, R4-6A2; BioLegend); Th2 conditions, IL-2 (10 ng ml^−1^), IL-4 (1 ng ml^−1^), and anti-IFN-γ mAb (5 μg ml^−1^, R4-6A2; BioLegend).

### Intracellular staining

For the intracellular staining of cytokines, cells were differentiated *in vitro* and stimulated with an immobilized anti-TCR-β mAb (3 μg ml^−1^, H57-597; BioLegend) for 6 h with monensin (2 μM, cat#M5273; Sigma-Aldrich), and intracellular staining was then performed^41^. For the intracellular staining of transcription factors, the cells tested without restimulation were stained using a transcription factor staining buffer kit according to the manufacturer's protocol (cat#TNB-0607-KIT; TONBO biosciences). Flow cytometry (fluorescence-activated cell sorting) was performed using a FACSCalibur instrument (BD Biosciences), and the results were analysed using the FlowJo software program (Tree Star).

### ELISA assay

Cells were stimulated with an immobilized anti-TCR-β mAb (3 μ ml^−1^) for 16 h. The concentrations of IL-2, IL-4, IL-5 and IFN-γ in the supernatants were determined using ELISAs, as described previously^41^. For the determination of IL-13, the DuoSet ELISA Kit (cat#DY413; R & D Systems) was used.

### Quantitative reverse transcriptase polymerase chain reaction

Total RNA was isolated using TRIzol reagent and complementary DNA (cDNA) was synthesized using the Superscript VILO cDNA synthesis kit (cat#11754; Life Technologies). Quantitative reverse transcription–PCR was performed using Step One Plus Real-Time PCR Systems (Life Technologies)^41^.

### Immunoprecipitaion and immunoblot analysis

Nuclear extracts were prepared using NE-PER Nuclear and Cytoplasmic Extraction Reagents (cat#78833; Thermo Fisher Scientific). In the case of immunoprecipitation with anti-Myc-Tag mAb (PL14), the cells were lysed with radioimmunoprecipitation buffer (1% nonidet P-40, 0.25% sodium deoxychorate, 150 mM NaCl, 1 mM EDTA, 1 mM phenylmethylsulphonyl fluoride, 1 μ ml^−1^ aprotinin, leupepton and pepstain, 1 mM Na_3_VO_4_, 1 mM NaF and 50 mM Tris-HCl (pH 7.4)). The lysates or precipitates were separated on an SDS polyacrylamide gel and then subjected to immunoblotting with specific antibodies. Images have been cropped for presentation. Full size images are presented in Supplementary Fig. 13.

### ChIP assay and ChIP-seq

The Magna ChIP kit was used for the ChIP assay according to the manufacturer's protocol (EMD-Millipore). Samples for ChIP-seq were prepared according to the manufacturer's protocol (Illumina) and sequenced using a Genome Analyser IIx device (Illumina). The obtained reads were mapped on the mouse reference genome sequence (mm9) using the Bowtie 0.12.7 software program. Peak calling was performed using the MACS 1.0.0. software program. In order to visualize the ChIP sequencing results, the data were converted to a wiggle file format at a 10-base resolution and were uploaded to the IGV platform (BROAD INSTITUTE, USA).

### Luciferase reporter assay

For the AP-1 promoter assay, the AP-1 luciferase reporter vector (BD Mercury Pathway Profiling Luciferase System, cat#631911) was used. Murine Bach2 cDNA was cloned into the pCMV-Myc vector (BD Bioscience). A luciferase assay of the AP-1 activity was performed using the 293 T cell line. The plasmids were transfected into 293 T cells using the GeneJammer Transfection Reagent (cat#204130; Agilent Technologies). Twenty-four hours after transfection, the cells were stimulated with 30 ng ml^−1^ of 4-beta-phorbol 12-myristate acetate for 16 h, and the luciferase activity was measured using a Dual-Luciferase Reporter Assay System (cat#E1910; Promega) according to the manufacturer's protocol.

### Oligonucleotide precipitation assay

The detailed protocol for the pull-down assay has been described previously^41^. Lysates were incubated with the indicated biotinylated oligonucleotides, and bound proteins were eluted and separated on an SDS polyacrylamide gel and then subjected to immunoblotting with specific antibodies. The oligonucleotide probes used for the pull-down assay were as follows:

AP-1 consensus: 5′ biotin- CGCTTGATGACTCAGCCGGAA -3′

AP-1 mutant: 5′ biotin- CGCTTGATGACTTGGCCGGAA -3′

Th2 LCR: 5′ biotin- AAGAGAAAGAAATGACTCAACATAAA -3′

Th2 LCR mutant: 5′ biotin- AAGAGAAAGAAATAAATAAACATAAA -3′

Th2#1 (*Il4* IE): 5′ biotin-GCATAGCCAAGTCATGTGT-3′

Th2#3 (RHS6): 5′ biotin- AGAAATGACTCAACATAAA -3′

Batf3#1: 5′ biotin-ATCTGTGACTCAGCTCCCC-3′

T-MARE: 5′ biotin- GGAATTGCTGACTCATTACT -3′

NF-E2: 5′ biotin- GCCTATGAGTCAGCAATTCC -3′

### Amplified luminescent proximity homogenous assay

AlphaScreen was performed as previously described using PerkinElmer's Kit^30^,^31^. In brief, Flag-tagged Bach2 protein was mixed with biotinylated transcription factors and incubated at 26 °C for 1 h. The mixture was then added to the detection mixture containing protein A-conjugated acceptor beads, streptavidin-coated donor beads and anti-Flag M2 antibody followed by incubation at 26 °C for 1 h. AlphaScreen signals from the mixture were detected using an EnVision device with the AlphaScreen signal detection program (PerkinElmer).

### Assessment of airway inflammation and hyperresponsiveness

BAL fluid cells and lung samples for the histological examination and RNA preparation were prepared as described previously^41^. BAL cells were cytocentrifuged onto slides and stained with Diff-Quik (cat#16920; sysmex). Two-hundred leukocytes were counted on each slide. Cell types were identified using morphological criteria. The percentages of each cell type were calculated. For lung histology, mice were killed by hyperanesthesia, and the lungs were infused with 4% (v/v) Formalin in PBS for fixation. The lung samples were sectioned, stained and examined for pathological changes under a light microscope. Airway hyperresponsiveness was assessed by measuring the changes in lung resistance (RL) and dynamic compliance (Cdyn) in response to increasing doses of inhaled methacholine, as described previously^41^.

### OVA-induced allergic inflammation in mice

Allergic airway inflammation was induced in mice as previously described^41^. In brief, WT and T cell-specific Bach2 TG mice were immunized intraperitoneally with 100 μg OVA in 2 mg of aluminum hydroxide gel on day 0. Next, the mice were intranasally challenged with OVA in saline (100 μg per mouse) on days 8, 9 and 10. Twenty-four hours after the last OVA challenge, BAL-fluid cells and lung sample were prepared for histological examination.

### Primers and probes for qPCR

The specific primers, and Roche Universal Probes were as follows: *Hprt*: 5′- TCCTCCTCAGACCGCTTT -3′ (forward), 5′- CCTGTTCATCATCGTAATC -3′ (reverse), probe #95; *18s*: 5′- GCAATTATTCCCCATGAACG -3′ (forward), 5′- GGGACTTAATCAACGCAAGC -3′ (reverse), probe #48; *Cd3ɛ*: 5′- AACACGTACTTGTACCTGAAAGCTC -3′ (forward), 5′- GATGATTATGGCTACTGCTGTCA -3′ (reverse), probe #10; *Bach2*: 5′- CAGTGAGTCGTGTCCTGTGC -3′ (forward), 5′- TTCCTGGGAAGGTCTGTGAT -3′ (reverse), probe #79; *Batf*: 5′- AGAAAGCCGACACCCTTCA -3′ (forward), 5′- CGGAGAGCTGCGTTCTGT -3′ (reverse), probe #85; *Batf3*: 5′- AGAAGGCTGACAAGCTCCAC -3′ (forward), 5′- CTGCGCAGCACAGAGTTC -3′ (reverse), probe #1; *Irf4*: 5′- ACAGCACCTTATGGCTCTCTG -3′ (forward), 5′- ATGGGGTGGCATCATGTAGT -3′ (reverse), probe #3; *Il21*: 5′- TCAGCTCCACAAGATGTAAAGG -3′ (forward), 5′- GCCTTCTGAAAACAGGCAAA -3′ (reverse), probe #100; *Clca3*: 5′- AGGAAAACCCCAAGCAGTG -3′ (forward), 5′- GCACCGACGAACTTGATTTT -3′ (reverse), probe #46; *Muc2*: 5′- CTTCAACGGCAGTCCAAAAT -3′ (forward), 5′- CTCAAGGGGTGTCAGCCTAA -3′ (reverse), probe #55.

The specific primers and Roche Universal probes used in ChIP-qPCR were as follows:

Th2 #1: 5′- GAAGCAGGGATGGTCAGACT -3′ (forward), 5′- TCCCCTAGCTATCCCCTAGC -3′ (reverse), probe #106; Th2 #2: 5′- TGGGACGACAGTGAGTCTGA -3′ (forward), 5′- CTTCCTGGCCAGAGAGTTTG -3′ (reverse), probe #71; Th2 #3 (RHS6): 5′- ATGCCTGCCCTAGCTACCTC -3′ (forward), 5′- CTTGCCTTCCTACCACTGGA -3′ (reverse), probe #94; Th2 #4: 5′- GCTTCCCTCCCTTTCCAG -3′ (forward), 5′- CCGACTTGGGGGTGAGTT -3′ (reverse), probe #106; IL-4 IE: 5′- CCCAAAGGAGGTGCTTTTATC -3′ (forward), 5′- AAATCCGAAACTGAGGAGTGC -3′ (reverse), probe #75; CGRE: 5′- CTCTCCTGGTGGCGTGTT -3′ (forward), 5′- CTTTGCGCACCCTTGAAC -3′ (reverse), probe #53; IL-5p: 5′- TCACTTTATCAGGAATTGAGTTTAACA -3′ (forward), 5′- GATCGGCTTTTCTTGAGCAC -3′ (reverse), probe #43; Batf #1: 5′- AAGTGGTTTGGAGAGCGAAA -3′ (forward), 5′- AAGCAAGCGCCTTTCACAT -3′ (reverse), probe #13; Batf #2: 5′- GGGTGCTGAGAATTGACCTC -3′ (forward), 5′- GCCTAGGCTGGTGAGACAGT -3′ (reverse), probe #46; Batf #3: 5′- CCAAGCAGATTAGGACCAAAA -3′ (forward), 5′- GACCAATGGCTTAGGCTTCC -3′ (reverse), probe #104; Batf #4: 5′- GTTGTTTCTCAGCAACTTCTATGC -3′ (forward), 5′- GTGGGGACTGCGTCATTT -3′ (reverse), probe #83; Batf #5: 5′- GCTGCCAAGCCTGTCAAT -3′ (forward), 5′- GCCAAGAAAATATAAGACAATGACC -3′ (reverse), probe #53; Batf3 #1: 5′- AATACGGTTTCCAGTGATTTCC -3′ (forward), 5′- ATGGAGTGTACTGTTCTAAAATGT -3′ (reverse), probe #95; Batf3 #2: 5′- GTGTGAGGACCGGGTGAG -3′ (forward), 5′- GATGAAACTGCCCACAGCA -3′ (reverse), probe #1; Batf3 #3: 5′- GCAGACAGCAAGTGAGTCAGA -3′ (forward), 5′- TGGGTTACAATGTGGGTGATT -3′ (reverse), probe #25; Batf3 #4: 5′- TAAAGGGTGAGCCTGAAACC -3′ (forward), 5′- CGATGCTGCTGCCTTTTAAC -3′ (reverse), probe #58; β-Actp: 5′- TCTTCTTGCAACACCTCCAG -3′ (forward), 5′- GCCATCCTATCCCAAGCATA -3′ (reverse), probe #45.

### Statistical analysis

Student's *t*-test, Welch's *t*-test, ANOVA and Bonferroni's test were used for the statistical analyses.

### Data availability

The ChIP-seq and DNA microarray data were deposited in the GEO database under the accession number database GSE65084. All other data presented in this article are available in the main and Supplementary Figures, or upon request from the authors.

## Additional information

**How to cite this article:** Kuwahara, M. *et al*. Bach2–Batf interactions control Th2-type immune response by regulating the IL-4 amplification loop. *Nat. Commun.* 7:12596 doi: 10.1038/ncomms12596 (2016).

## Supplementary Material

Supplementary Information Supplementary Figures 1-13
