# Supplementary material for: Bach2–Batf interactions control Th2-type immune response by regulating the IL-4 amplification loop
Source: Nat Commun. 2016 Sep 1;7:12596. doi: 10.1038/ncomms12596 (PMC5025763; doi:10.1038/ncomms12596)
Supplement: Supplementary Information — Supplementary Figures 1-13 [file ncomms12596-s1.pdf]

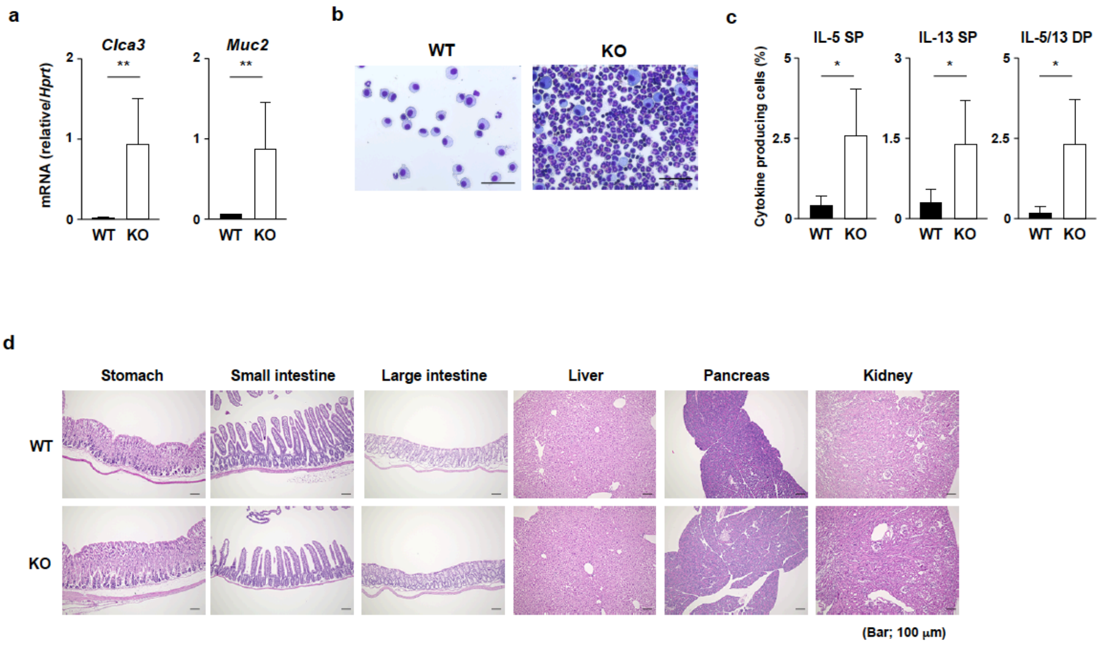

Supplementary Fig. 1. Spontaneous development of lung inflammation in T cell-specific *Bach2*-deficient mice

(a) The results of the quantitative RT-PCR analysis of the *Clca3* and *Muc2* mRNA in the lungs of WT control and *Bach2*$^{fl/fl}$ × CD4-Cre TG (*Bach2*-deficient) mice (mean ±SD, n = 8). The results are presented relative to the expression of *Hprt* mRNA with the standard deviation. ***P*< 0.01 (Student's *t*-test). **(b)** Diff-Quick staining of the BAL fluid cells derived from WT control and *Bach2*-deficient mice. (Scale bars = 50 μm). The results are representative of eight independent experiments. **(c)** The percentages of IL-5- and IL-13-producing cells in the lung CD4 T cells of WT control and *Bach2*-deficient mice are shown with the standard deviation (mean ±SD, n = 5). **P*< 0.05 (Student's *t*-test). **(d)** Microscopic findings of the indicated organs derived from WT and *Bach2*-deficient mice (n = 5 per group), fixed and stained with

hematoxylin and eosin. The results are representative of three independent experiments.

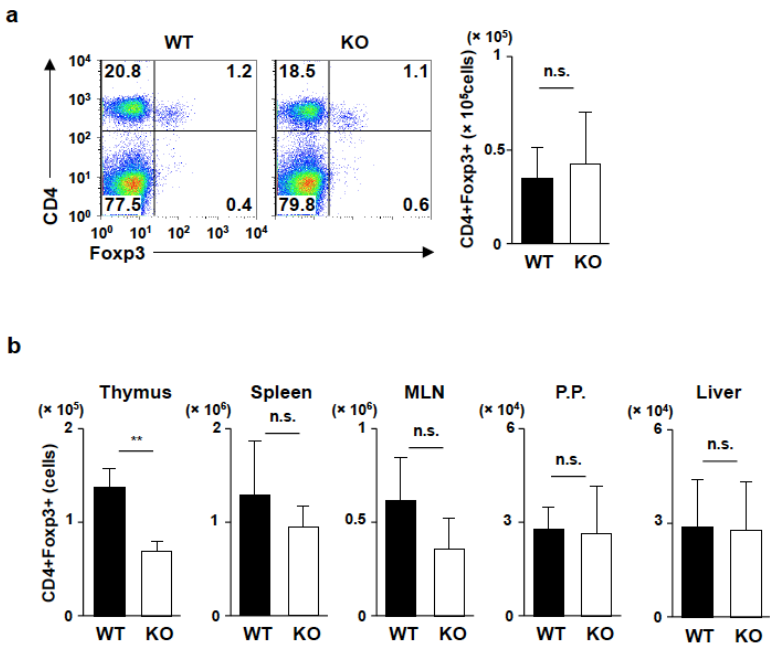

Supplementary Fig. 2. Differentiation of regulator T cells in T cell-specific *Bach2*-deficient mice

(a) The results of the intracellular flow cytometry analysis of Foxp3 in the lung CD4 T cells of *Bach2*-deficient mice. The cell numbers of Foxp3-positive CD4 T cells in the lungs are shown with standard deviations (mean ±SD, n = 5; right panel). **(b)** The numbers of Foxp3-positive CD4 T cells in the thymus, spleen, mesenteric lymph nodes, Peyer's patch (P.P.) and liver are shown with standard deviations (mean ±SD, n = 5). ***P*< 0.01, n.s.: not significant (Student's *t*-test).

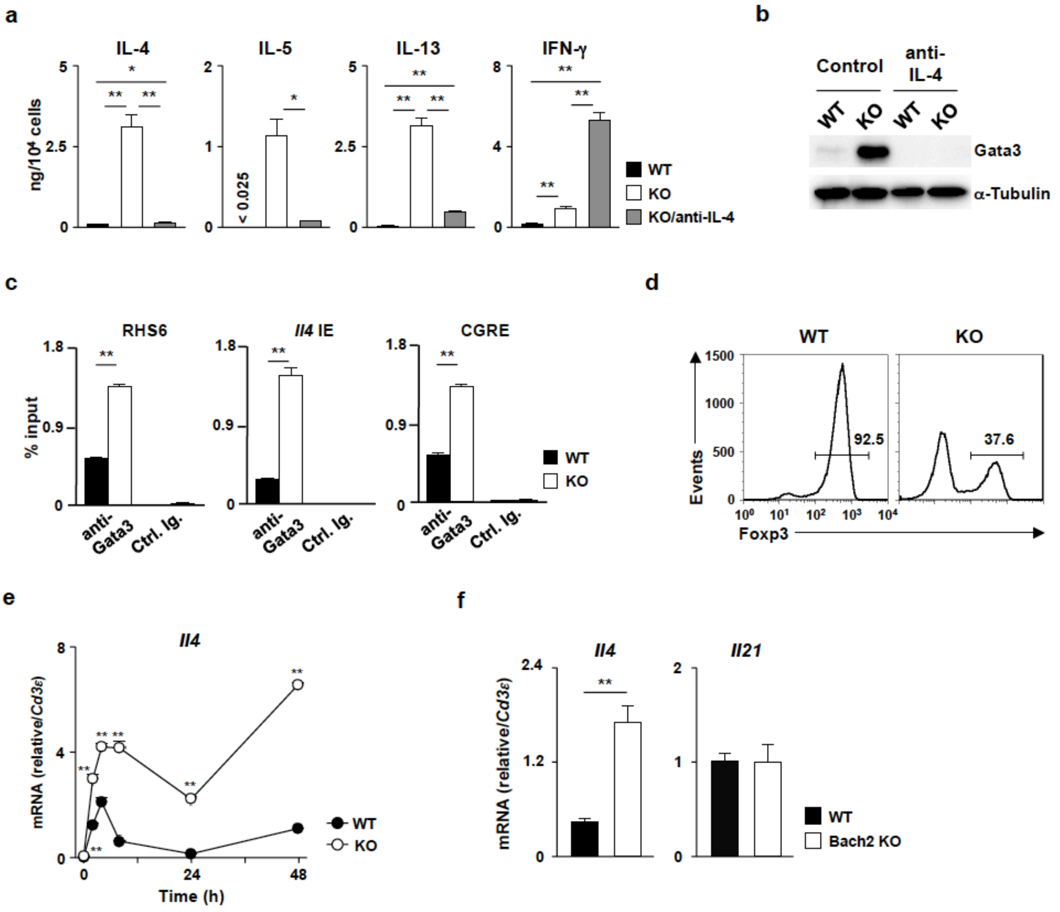

Supplementary Fig. 3. Enhanced Th2 cell differentiation in *Bach2*-deficient T cells

(a) The results of the ELISA for cytokines in the supernatants of the cells in (**Fig. 2a**) stimulated with an immobilized anti-TCR-β mAb for 16 hours. **P< 0.01 (Student's t-test) (mean ±SD, n = 3). **(b)** The results of the immunoblot analysis of Gata3 in the nuclear fractions of WT or *Bach2*-deficient naïve CD4 T cells cultured under IL-2 or neutral conditions for four days. The results of an analysis with an antibody against α-tubulin (Anti-α-tubulin) served as a loading control for the cytosolic fractions. **(c)** The results of the ChIP assay of the binding of Gata3 to the Rad50 hypersensitive site 6

(RHS6) within the locus control region (LCR) of the Th2 cytokine gene locus, the *Il4* intron enhancer (*IL-4* IE) and the Conserved Gata response element (CGRE) in naïve CD4 T cells cultured under IL-2 conditions for two days; the results are presented relative to those of input DNA with the standard deviation. $^{**}P < 0.01$ (Student's *t*-test) (mean ±SD, n = 3). **(d)** Results of the intracellular flow cytometry analysis of Foxp3 expression in the WT and *Bach2*-deficient (KO) naïve CD4 T cells cultured under iTreg conditions for 3 days. The ratio of cells is indicated in each quadrant. The results are representative of three independent experiments. (**e**) The kinetics of the *Il4* mRNA expression in WT and *Bach2*-deficient naïve CD4 T cells cultured under IL-2 conditions for the indicated times. The results are presented relative to the mRNA expression of *Cd3ε* with the standard deviation (mean ±SD, n = 3). $^{**}P < 0.01$ (Student's *t*-test). **(f)** The results of the quantitative RT-PCR analysis of *Il4* and *Il21* mRNA in *Bach2*-deficient Tfh cells isolated from Peyer's patch. The $CD3\varepsilon^{+}CD4^{+}PD1^{high}CXCR5^{high}$ cells were sorted by flow cytometry, and then stimulated with an anti-TCRβ mAb for 4 hours. The results are presented relative to the mRNA expression of *Cd3ε*, with the standard deviation. $^{**}P < 0.01$ (Student's *t*-test) (mean ±SD, n = 3).

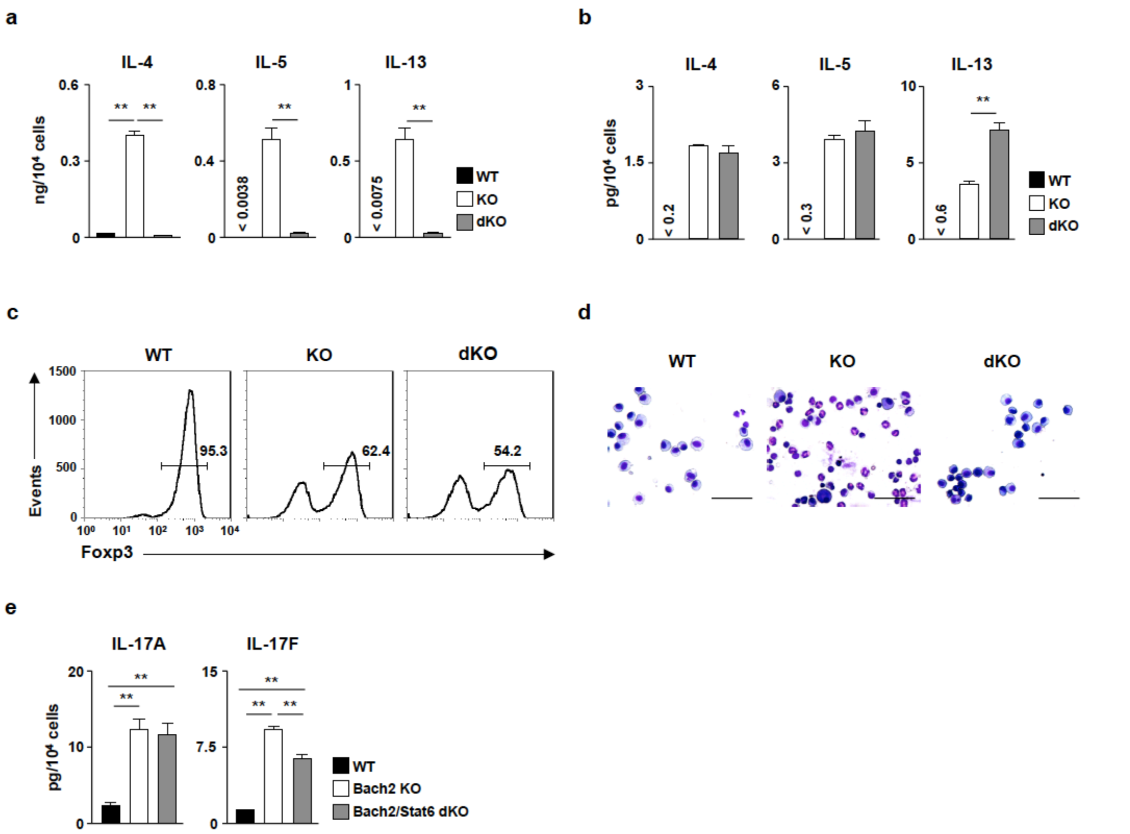

Supplementary Fig. 4. Phenotypic characterization of *Bach2*/*Stat6* double-deficient CD4 T cells

(a) The results of the ELISA for cytokines in the supernatants of the cells in (**Fig. 2c**) stimulated with an immobilized anti-TCR-β mAb for 16 hours. **P< 0.01 (Student's *t*-test) (mean ±SD, n = 3). **(b)** The results of the ELISA for cytokines in the supernatants of the WT, *Bach2*-deficient (KO) and *Bach2*/*Stat6* double-deficient (dKO) naïve CD4 T cells stimulated with an immobilized anti-TCR-β mAb and an anti-CD28 mAb for 16 hours. **P< 0.01 (Student's *t*-test) (mean ±SD, n = 3). **(c)** The results of the intracellular flow cytometry analysis of Foxp3 expression in the WT, *Bach2*-deficient (KO) and *Bach2*/*Stat6* double-deficient (dKO) naïve CD4 T cells cultured under iTreg conditions for 3 days. The ratio of cells is indicated in each

quadrant. The results are representative of three independent experiments. **(d)** Diff-Quick staining of the BAL fluid cells derived from WT control, *Bach2*-deficient and *Bach2*/*Stat6* double-deficient mice. (Scale bars = 50 μm). The results are representative of five independent experiments. **(e)** The results of the ELISA for IL-17A and IL-17F in the supernatants derived from the lung CD4 T cells stimulated with an immobilized anti-TCR-β mAb and an anti-CD28 mAb for 16 hours. The production level of IL-17A and IL-17F from WT, *Bach2* KO and *Bach2/Stat6* double KO lung CD4 T cells was determined. $^{*}P < 0.05$, $^{**}P < 0.01$ (Student's *t*-test) (mean ±SD, n = 3).

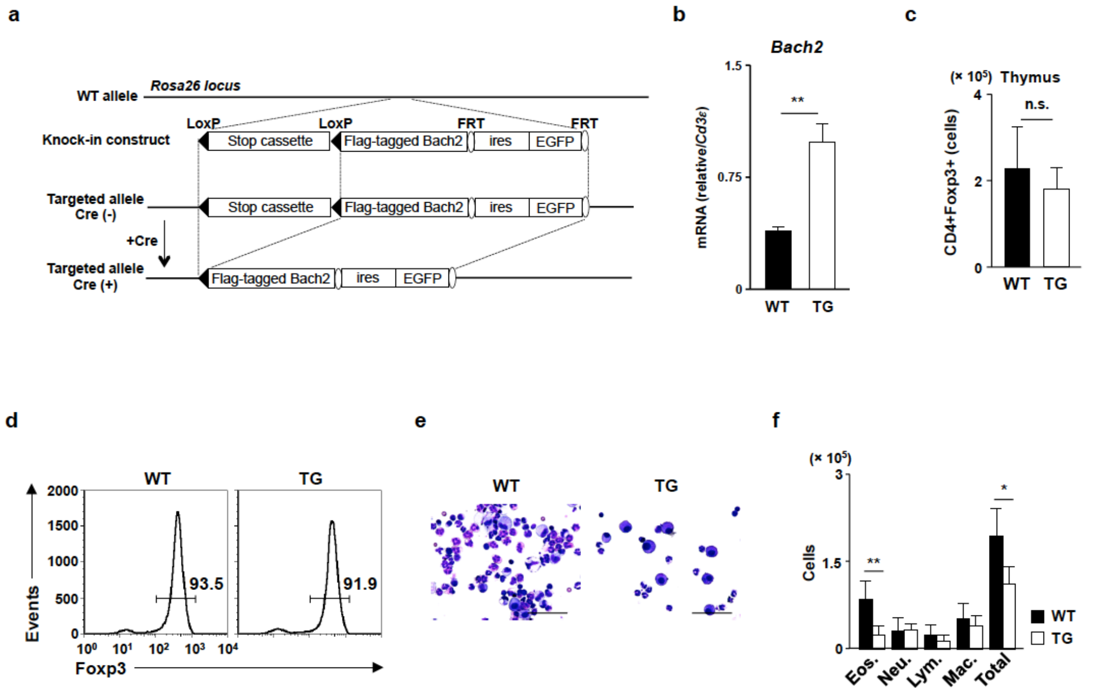

Supplementary Fig. 5. Phenotypic characterization of T cell-specific *Bach2* TG mice

(a) A schematic diagram of the Rosa26-stop-Flag-Bach2 knock-in mice (conditional *Bach2* TG mice). The conditional *Bach2* TG mice were crossed with CD4 Cre TG mice to generate T cell-specific *Bach2* TG mice. The heterozygote *Bach2* TG mice were used for analysis. **(b)** The expression of *Bach2* mRNA in effector CD4 T cells from Rosa26-Stop cassette$^{flox/flox}$-Bach2 TG x CD4-Cre TG mice. WT and Bach2 TG naïve CD4 T cells were cultured under Th2 condition for 5 days. The results are presented relative to the mRNA expression of *Cd3ε*, with the standard deviation. ***P*< 0.01 (Student's *t*-test) (mean ±SD, n = 3). **(c)** Foxp3-positive CD4 T cells in the thymus of *Bach2*-TG mice are shown with the standard deviation (mean ±SD, n = 3). n.s.: not significant (Student's *t*-test) **(d)** The results of the intracellular flow

cytometry analysis of Foxp3 expression in the WT and *Bach2*-TG naïve CD4 T cells cultured under iTreg conditions. The ratio of cells is indicated in each quadrant. The results are representative of three independent experiments. **(e)** Diff-Quick staining of the BAL fluid cells derived from OVA-immunized WT control and Bach2 TG mice. (Scale bars = 50 μm). **(f)** The absolute number of eosinophils (Eos.), neutrophils (Neu.), lymphocytes (Lym.), macrophages (Mac.) and total cells (Total) are shown with the standard deviation in the BAL-fluid from the OVA-challenged WT or Bach2 TG mice (mean ±SD, WT; n = 4, TG; n = 5) immunized with OVA. *$P < 0.05$, **$P < 0.01$ (Student's *t*-test).

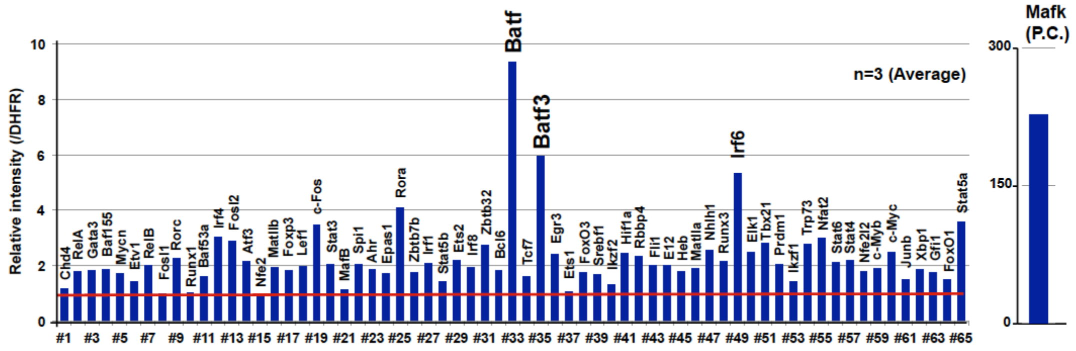

Supplementary Fig. 6. Results of the AlphaScreen to identify the Bach2 interaction transcriptional regulators

Sixty-five transcriptional regulators were synthesized by cell-free technology and determined the interaction with Bach2 using AlphaScreen. The relative intensities to Dihydrofolate reductase (DHFR) binding are presented as the averages of three independent experiments. The red line indicates the basal interaction. The interaction of Bach2 with MafK is indicated in a positive control (right).

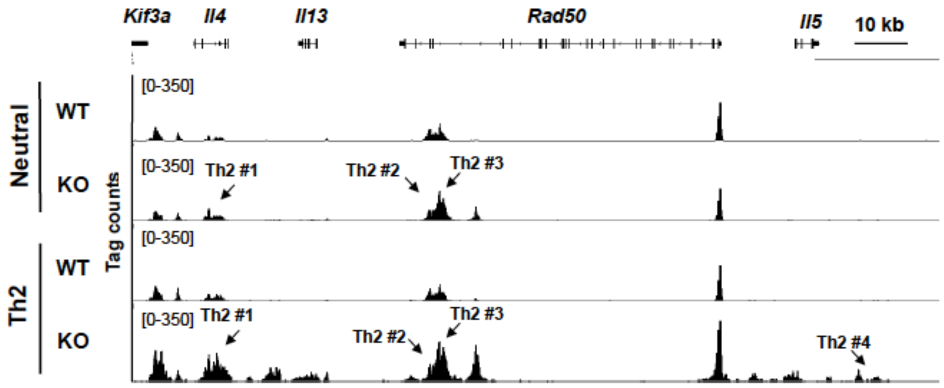

Supplementary Fig. 7. Global patterns of histone H3K27 acetylation at the Th2 cytokine gene loci in the WT and *Bach2*-deficient naïve CD4 Tcells

Naïve CD4 T cells were cultured under neutral conditions for five days were determined using a ChIP-seq analysis.

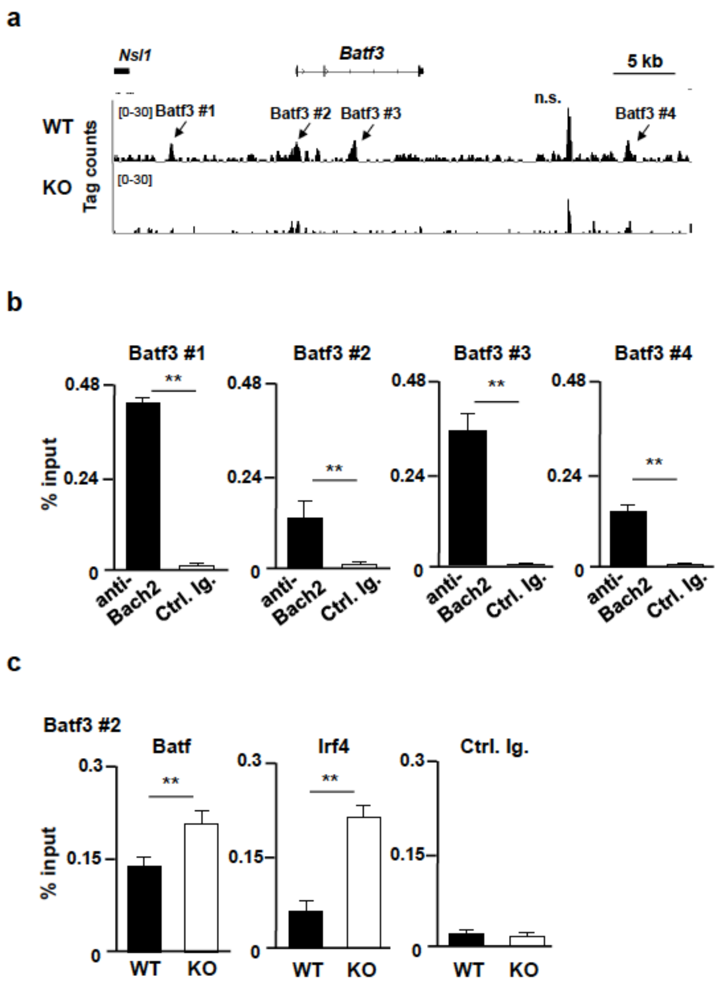

Supplementary Fig. 8. The binding of Bach2 to the *Batf* gene locus

(a) The global patterns of Bach2 binding at the *Batf3* gene locus in the WT and *Bach2*-deficient naïve cells cultured under neutral conditions for five days were determined using ChIP-seq. **(b)** Results of the ChIP assay with a quantitative PCR analysis of Bach2 binding in the WT naïve CD4 T cells cultured under IL-2 conditions for 48 hours; the results are presented relative to those of input DNA with the standard deviation. $^{**}P< 0.01$ (Student's *t*-test) (mean ±SD, n = 3). **(c)** Binding of Batf and Irf4 at the Batf3#2 region was determined using a ChIP assay with quantitative PCR. The results are presented relative to those of input DNA with the standard deviation.

**$P < 0.01$ (Student's *t*-test) (mean ±SD, n = 3).

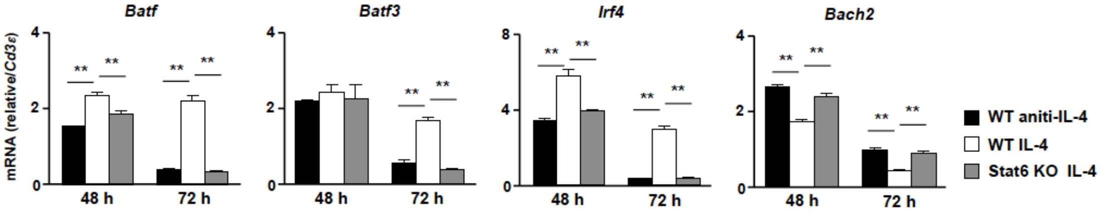

Supplementary Fig. 9. Results of a quantitative RT-PCR analysis of *Bach2*, *Batf*, *Irf4* and *Bach2* mRNA

WT or *Stat6*-deficient naive CD4 T cells stimulated with an anti-TCRβ mAb plus an ant-CD28 mAb in the presence or absence of IL-4 for the indicated hours. The results are presented relative to the mRNA expression of *Cd3ε*, with the standard deviation. $^{**}P < 0.01$ (Student's *t*-test) (mean ±SD, n = 3).

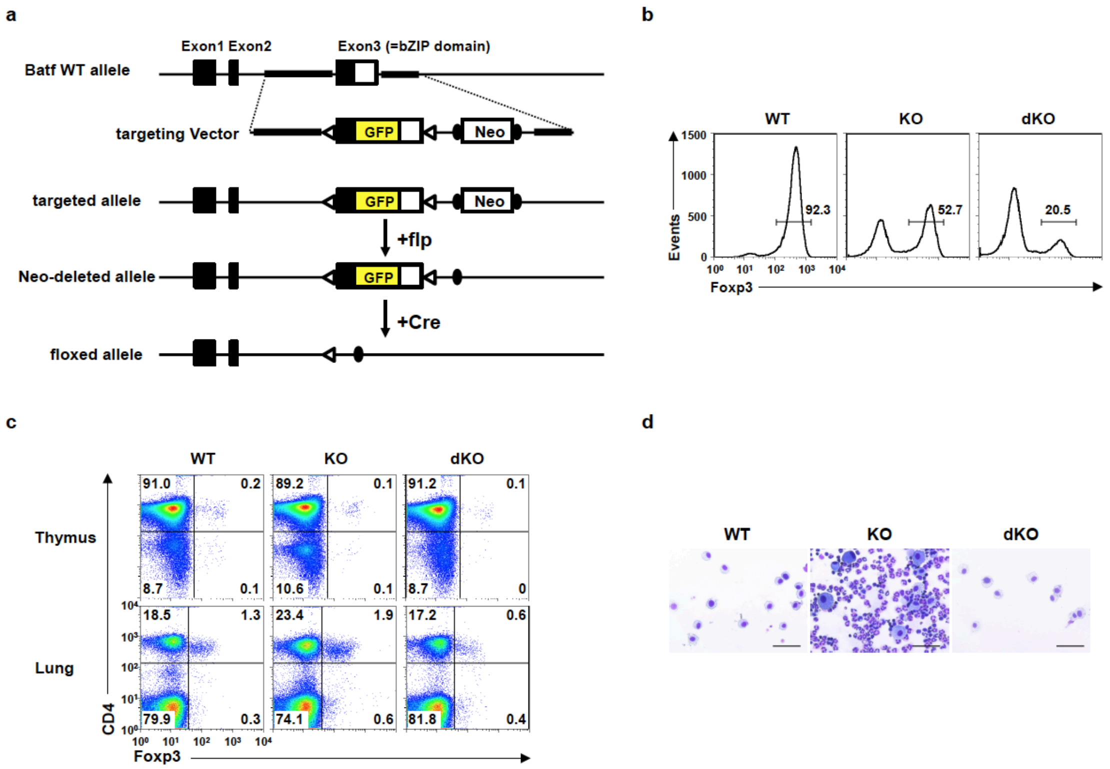

Supplementary Fig. 10. Phenotypic characterization of T cell-specific *Bach2*/*Batf* double-deficient CD4 T cells

(a) A schematic diagram of the *Batf*-floxed allele. The *Batf*-floxed mice were crossed with CD4 Cre TG mice to generate T cell-specific *Batf*-deficient mice. **(b)** The results of the intracellular flow cytometry analysis of Foxp3 expression in the WT, *Bach2*-deficient (KO) and *Bach2*/*Batf* double-deficient (dKO) naïve CD4 T cells cultured under iTreg conditions for 3 days. The ratio of cells is indicated in each quadrant. The results are representative of three independent experiments. **(c)** The results of the intracellular flow cytometry analysis of Foxp3-positive CD4 T cells in the thymus and lungs of WT, *Bach2*-deficient (KO) and *Bach2*/*Batf* double-deficient (dKO) mice. The results are representative of three independent experiments. **(d)**

Diff-Quick staining of the BAL fluid cells derived from WT control, *Bach2*-deficient and *Bach2*/*Batf* double-deficient mice. (Scale bars = 50 μm).

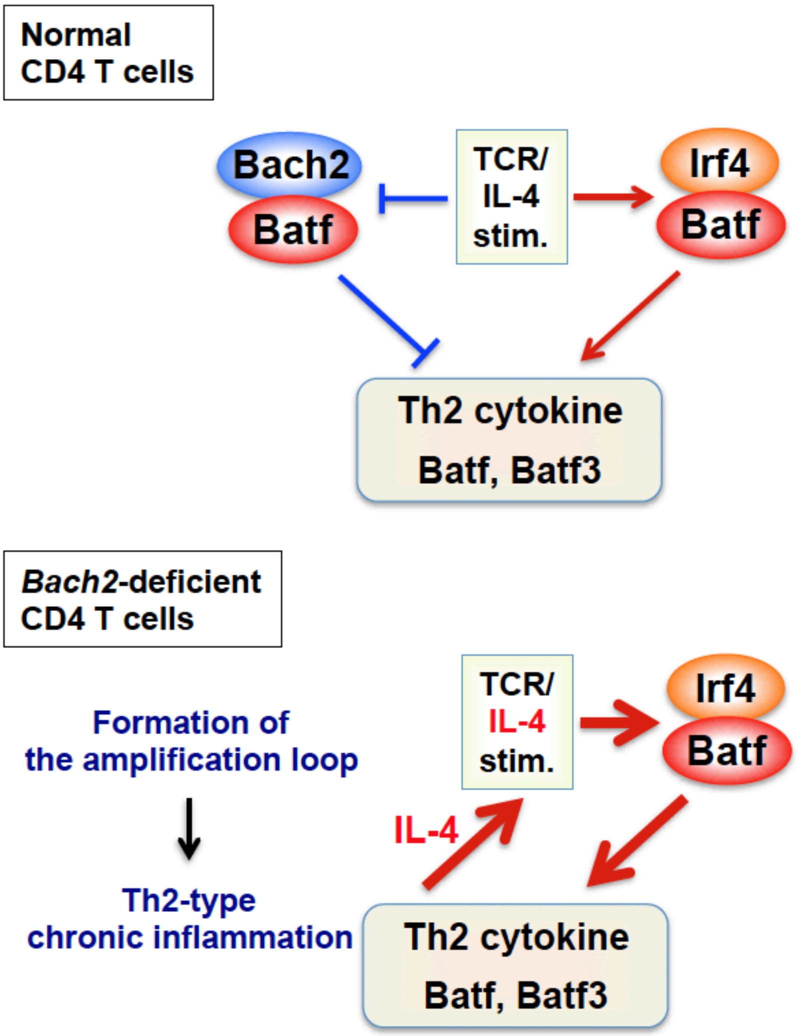

Supplementary Fig. 11. A schematic representation of Bach2-mediated regulation of Th2-type immune response

The Bach2-Batf interactions inhibit the formation of an IL-4 amplification loop to induce Th2 cell development and the subsequent onset of Th2-type inflammation.

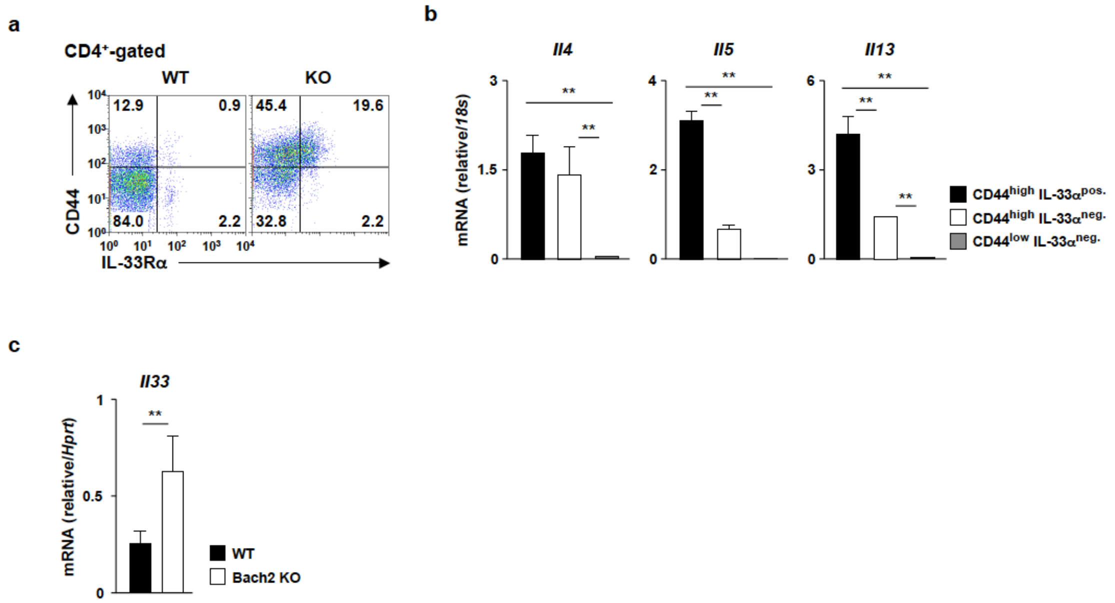

Supplementary Fig. 12. Increased number of IL-33Rα-expressing lung CD4 T cells in T cell-specific *Bach2*-deficient mice

(a) The results of the flow cytometry analysis of CD44 and IL-33Rα in lung CD4 T cells. The numbers of cells are indicated in each quadrant. **(b)** The results of a quantitative RT-PCR of *Il4*, *Il5* and *Il13* mRNA in the indicated fractions of lung CD4 T cells from *Bach2*-deficient mice. The cells were sorted by FACS and stimulated with an anti-TCRβ mAb and an anti-CD28 mAb for 4 hours. The results are presented relative to the *18s* ribosomal RNA. $^{**}P < 0.01$ (Student's *t*-test) (mean ±SD, n = 3). **(c)** The result of the quantitative RT-PCR analysis of *Il33* mRNA in the lungs of *Bach2*-deficient mice. The results are presented relative to the *Hprt* mRNA. $^{**}P < 0.01$ (Student's *t*-test) (mean ±SD, n = 5).

Fig. 4d

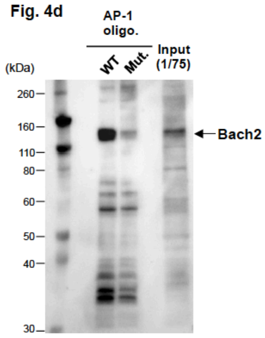

Fig. 5a upper (i.p.: Myc)

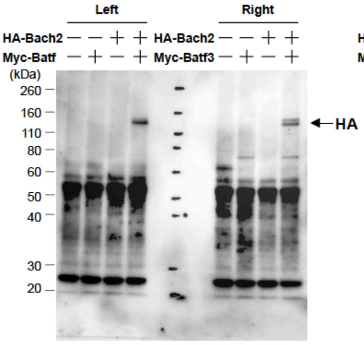

Fig. 5a middle (input)

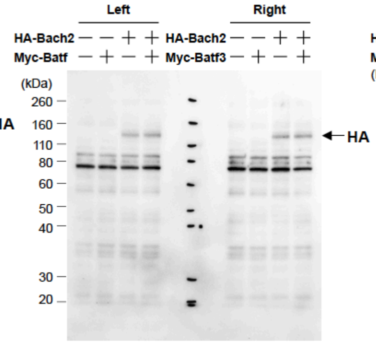

Fig. 5a lower (input)

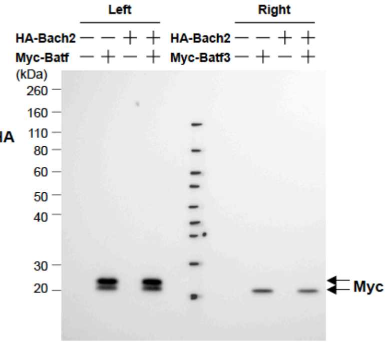

Fig. 5b pull down

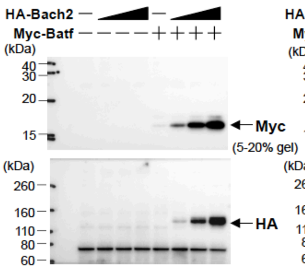

Fig. 5b input

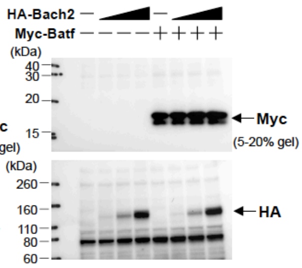

Fig. 5c upper

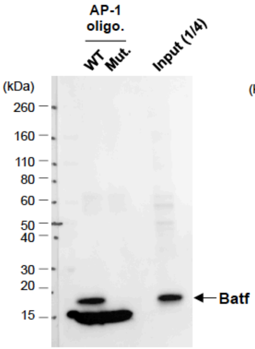

Fig. 5c lower

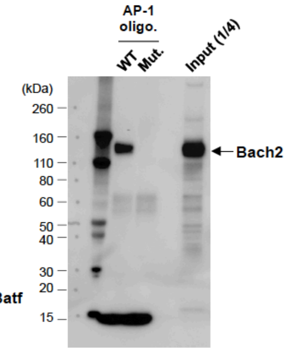

Fig. 5e left upper

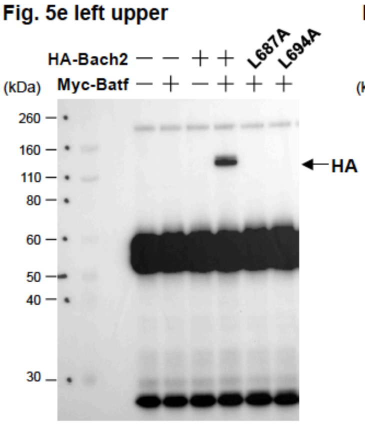

Fig. 5e left middle

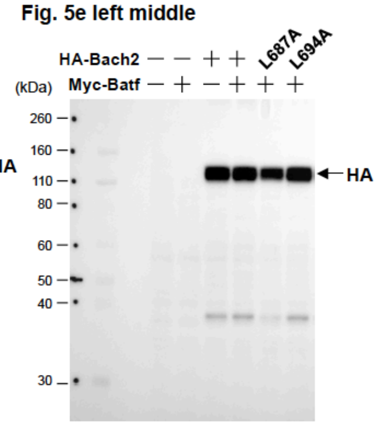

Fig. 5e left lower

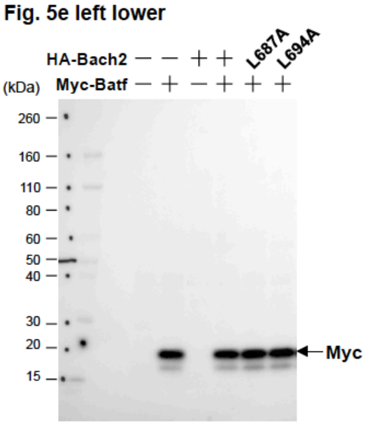

Fig. 5e right upper

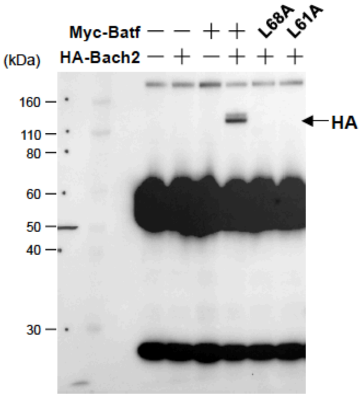

Fig. 5e right middle

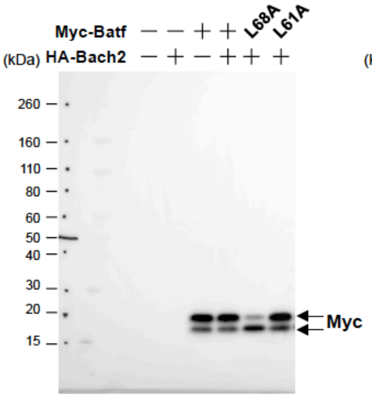

Fig. 5e right lower

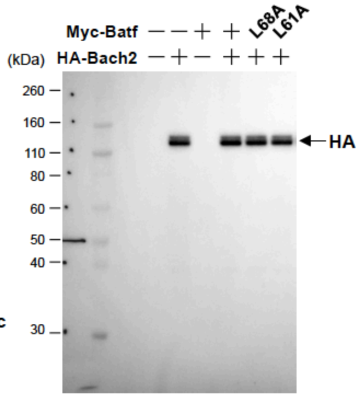

Fig. 6d upper

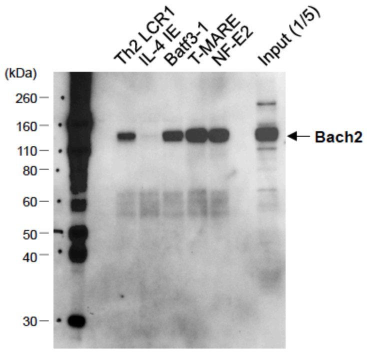

Fig. 6d middle

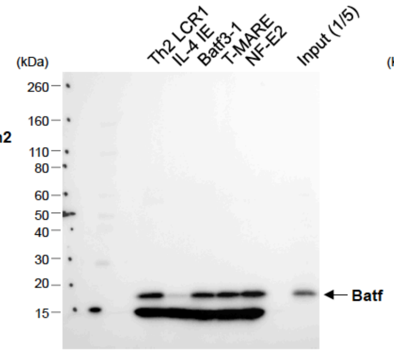

Fig. 6d lower

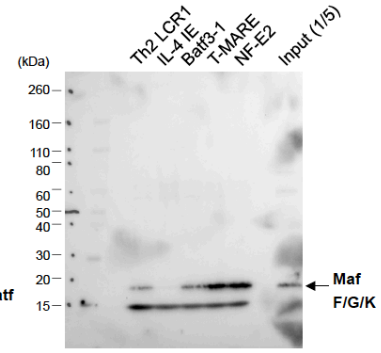

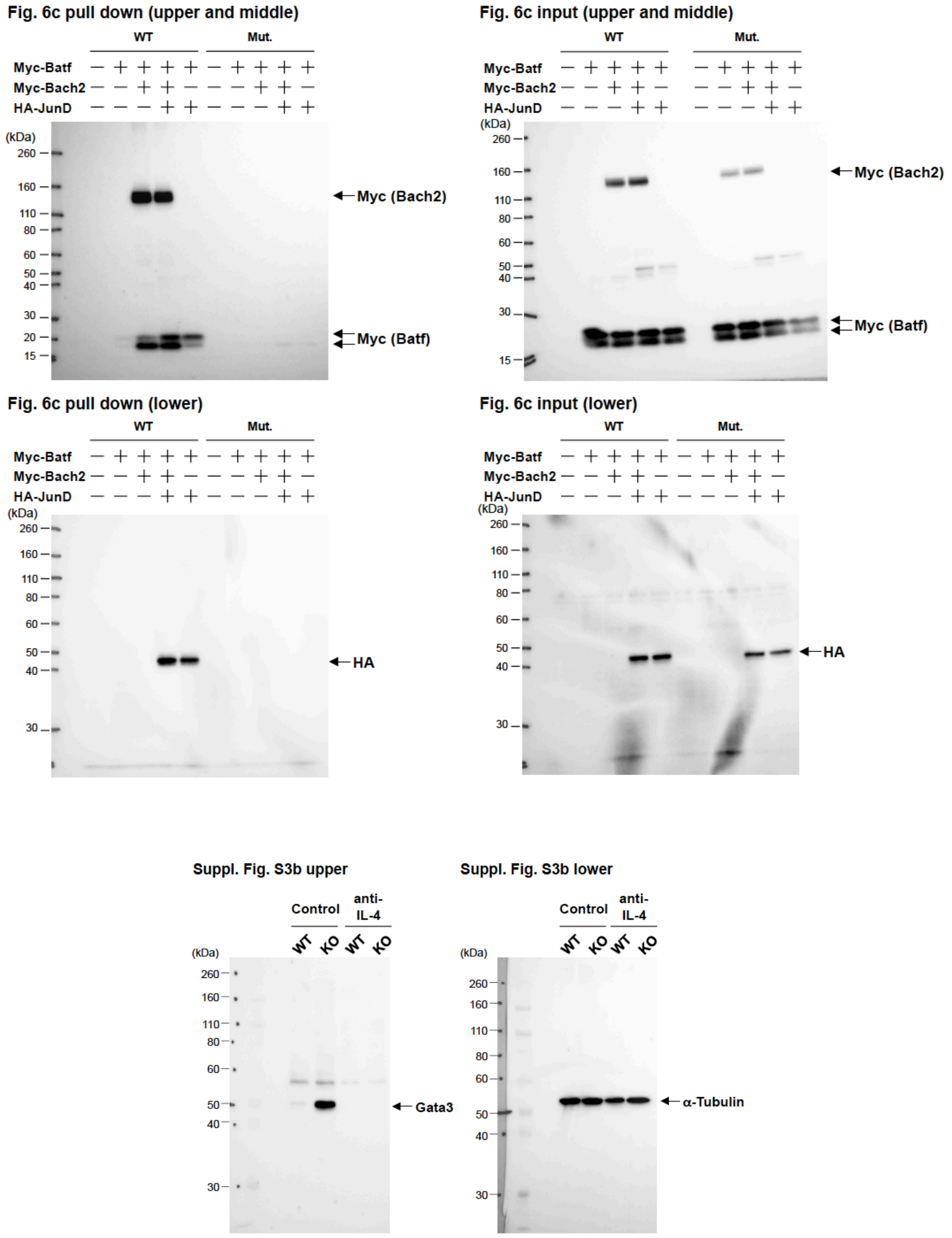

Supplementary Fig. 13. Full-size scans of all western blots are shown with labeled lanes and molecular weight ladder.
